# Modeling the influence of stromal microenvironment in the selection of ENU-induced BCR-ABL1 mutants by tyrosine kinase inhibitors

**DOI:** 10.18632/oncoscience.9

**Published:** 2014-01-30

**Authors:** Djamel Aggoune, Lucie Tosca, Nathalie Sorel, Marie-Laure Bonnet, Fatima Dkhissi, Gérard Tachdjian, Annelise Bennaceur-Griscelli, Jean-Claude Chomel, Ali G Turhan

**Affiliations:** ^1^ INSERM, U935, F-86000 Poitiers, France; ^2^ INSERM, U935, F-94800, Villejuif, France; ^3^ Université Paris–Sud 11, F-94270 Le Kremlin-Bicêtre, France; ^4^ Hôpital Antoine Béclère, Service d'Histologie-Embryologie-Cytogénétique, F-92140 Clamart, France; ^5^ CHU de Poitiers, Service de Cancérologie Biologique, F-86000 Poitiers, France; ^6^ Hôpital Paul Brousse, Service d'Hématologie Biologique, F-94800 Villejuif, France; ^7^ Hôpital Bicêtre, Service d'Hématologie Biologique, F-94270 Le Kremlin Bicêtre, France

**Keywords:** ENU-mutagenesis, BCR-ABL1, CML, microenvironment, TKI resistance

## Abstract

Tyrosine kinase inhibitors (TKIs) have profoundly changed the natural history of chronic myeloid leukemia (CML). However, acquired resistance to imatinib, dasatinib or nilotinib (1^st^ and 2^nd^ generation TKIs), due in part to BCR-ABL1 kinase mutations, has been largely described. These drugs are ineffective on the T315I gatekeeper substitution, which remains sensitive to 3^rd^ generation TKI ponatinib. It has recently been suggested that the hematopoietic niche could protect leukemic cells from targeted therapy. In order to investigate the role of a stromal niche in mutation-related resistance, we developed a niche-based cell mutagenesis assay. For this purpose, ENU (N-ethyl-N-nitrosourea)-exposed UT-7 cells expressing non-mutated or T315I-mutated BCR-ABL1 were cultured with or without murine MS-5 stromal cells and in the presence of imatinib, dasatinib, nilotinib, or ponatinib. In the assays relative to 1^st^ and 2^nd^ generation TKIs, which were performed on non-mutated BCR-ABL1 cells, our data highlighted the increasing efficacy of the latter, but did not reveal any substantial effect of the niche. In ponatinib assays performed on both non-mutated and T315I–mutated BCR-ABL1 cells, an increased number of resistant clones were observed in the presence of MS-5. Present data suggested that T315I mutants need either compound mutations (e.g. E255K/T315I) or a stromal niche to escape from ponatinib. Using array-comparative genomic hybridization experiments, we found an increased number of variations (involving some recurrent chromosome regions) in clones cultured on MS-5 feeder. Overall, our study suggests that the hematopoietic niche could play a crucial role in conferring resistance to ponatinib, by providing survival signals and favoring genetic instability.

## INTRODUCTION

Chronic myeloid leukemia (CML) is a paradigm of leukemogenesis initiated by the appearance of the Philadelphia (Ph1) chromosome in a primitive hematopoietic stem cell (HSC) [1]. The *BCR-ABL1* fusion gene, which is the counterpart of the Ph1 chromosome, gives rise to the p210^*BCR-ABL1*^ protein characterized by deregulated tyrosine kinase activity. It is thought to be responsible for the phenotypic features of the disease, including genetic instability [2]. The ability to target this tyrosine kinase protein by the use of small inhibitors is challenging since BCR-ABL1 activates a plethora of signaling pathways [3]. In this context, imatinib, which showed selective inhibitory activity with regard to BCR-ABL1, was the first TKI (tyrosine kinase inhibitor) developed and tested successfully in patients to become the standard front-line treatment of chronic phase CML [4,5]. However, up to 20-30% of patients develop resistance towards imatinib. This phenomenon can be either oncogene-dependent (e.g. BCR-ABL1 amplification or mutations), or –independent (e.g. activation of SRC kinase families) [6].

Point mutations occurring within the BCR-ABL1 kinase domain (KD) have become the most widely known mechanism of imatinib resistance. Up until now, over 100 mutations affecting 70 amino acids have been described [7]. In order to efficiently target these mutants, second-generation TKIs have been developed. Nilotinib, of which the design was based on imatinib, binds to BCR-ABL1 with better efficacy [8]. Dasatinib, which was developed first as a SRC inhibitor, can bind the BCR-ABL1 KD regardless of the activation loop conformation [9]. Just like nilotinib, it is more potent than imatinib but is less selective than either. Second-generation TKIs are currently used in clinical practice and are efficient on most of the mutants, with the exception of the threonine-isoleucine substitution at position 315 (T315I) [10]. More recently, ponatinib, considered as a pan-BCR-ABL1 inhibitor, was shown to be active against T315I mutants [11].

It is now well established that primitive HSCs are refractory to all TKIs used in clinical practice [12-14]. This resistance to TKIs can be acquired through different mechanisms, but a close relation between leukemic stem cells (LSCs) and the bone marrow microenvironment could play a particularly important role [15,16]. The stem cell niche can provide survival and/or quiescence signals to LSCs and favor the persistence of a pool of residual leukemic clones comprising mutants.

The objective of the present work was to apprehend the potential influence of the microenvironment in the emergence of BCR-ABL1 KD mutants in the presence of TKI. For that purpose, we developed a niche-based cell mutagenesis assay using UT-7 cells expressing native or T315I mutated BCR-ABL1 (as CML models) and the murine stromal cell line MS-5 (as a niche model). This cell line creates a surrogate microenvironmental niche that can promote the expansion or differentiation of human HSCs *in vitro*. Using this screening protocol, four TKIs (imatinib, nilotinib, dasatinib and ponatinib) were tested. TKI-resistant UT-7 clones, appearing in the presence or in the absence of the niche, were analyzed for BCR-ABL1-kinase mutations, and some of them were further analyzed by array-comparative genomic hybridization (array-CGH). The present strategy highlighted a potential role of the stromal niche in the acquired resistance of T315I mutants towards ponatinib.

## RESULTS

### Mutagenesis assay in UT-7 cells and TKI selection

Our assay was based on human UT-7 cell lines (expressing non-mutated or T315I-mutated BCR-ABL1), mutagenized with ENU (N-ethyl-N-nitrosourea) and cultured with or without murine MS-5 stromal cells in the presence of imatinib, dasatinib, nilotinib, or ponatinib (Supplementary Fig. S1). UT-7 cell lines were characterized with regard to BCR-ABL1 expression by western blots. As expected, the BCR-ABL1 oncoprotein was detected in UT-7-11 and UT-7-315, but not in parental UT-7 cell line from which they were derived (Supplementary Fig. S2A). In addition, mutation screening of the BCR-ABL1 KD domain by DGGE (denaturing gradient gel electrophoresis) confirmed the presence of the gatekeeper mutation in UT-7-315 and the absence of any mutation in UT-7-11 or in parental UT-7 cell line (Supplementary Fig. S2B).

To establish the optimal ENU concentration, we tested four different doses (25, 50, 75 and 100 μg/ml) and determined cell viability using a trypan blue exclusion assay after 24h of culture. With mortality exceeding 50-60%, concentrations higher than 50μg/ml appeared to be very toxic for UT-7 cells, whereas an acceptable toxicity was achieved with 50 μg/ml (20%). A pilot study, with 24 clones, was then performed to verify the ability of ENU to induce mutagenesis at this concentration. All the clones tested were mutated and harbored at least one mutation in the BCR-ABL1 KD domain. Because 50μg/ml ENU was shown to be efficient without high toxicity, this dose was subsequently used in culture experiments.

In order to determine the concentration of each TKI used in the mutagenesis assay, UT-7 parental cell line and BCR-ABL1-expressing counterparts were tested for cell viability after 72 hours of exposure using trypan blue exclusion assay. For each TKI, a range of concentrations was tested on all UT-7 cell lines, and the concentration corresponding to the beginning of the plateau was retained as effective (Supplementary Table S1). In the presence of TKIs, due to the lack of the BCR-ABL1 oncogene, UT-7 parental cells displayed minimal cell death. All 4 TKIs appeared active on UT-7-11 cells expressing non-mutated BCR-ABL1 since elevated cell death percentage was observed in all conditions. As expected, only ponatinib caused cell death on 86% of T315I mutated BCR-ABL1 expressing cells (UT-7-315). For optimal selection pressure, the working TKI concentrations chosen were higher than the effective dose; i.e. 2μM for imatinib, 75nM for nilotinib, 10nM for dasatinib and 30nM for ponatinib.

### Outgrowth of TKI-resistant clones: Effect of the MS-5 niche

The first objective of the present screening was to evaluate the potential role of MS-5 feeder cells on the emergence of TKI-resistant UT-7 clones after ENU treatment. With that in mind, for each TKI (imatinib, nilotinib, dasatinib or ponatinib), UT-7-11 mutagenized cells were cultured in two 96-well plates with or without MS-5 feeder cells. To mimic the clinical condition in which a patient carrying the gatekeeper mutation is treated with ponatinib, the ENU-mutagenesis test was performed on UT-7-315 cells. During this assay, experiments were conducted in duplicate (2 plates with MS-5 and 2 plates without). UT-7 cell growth was evaluated by microscopic inspection of each well twice a week for 5-6 weeks. A well was considered as positive when cell growth was observed in the presence of the drug. In the absence of viable UT-7 cells, the well was considered as negative. Positive and negative wells were enumerated in all plates in order to determine, after TKI selection, the number and percentage of TKI-resistant clones with or without MS-5 feeder.

Table 1 summarizes the results of these experiments. The number of imatinib-resistant UT-7 clones was similar when the mutagenized cells were grown in the absence (56%) or in the presence (53%) of MS-5. Similarly, dasatinib selection yielded similar percentages of UT-7 resistant clones in the absence (12.5%) or in the presence (11.5%) of the stromal niche. Using the same experimental conditions and nilotinib, no outgrowth of UT-7-11 resistant clones with MS-5 feeder was observed. Nevertheless, viable cells located within the MS-5 feeder were detected by meticulous microscopic inspection. To confirm the presence of these UT-7-11 clones, 24 wells were randomly selected and replated in methylcellulose, in the presence of 75nM nilotinib. After culture for 14 days, hematopoietic colonies were recovered in all cases, thereby confirming the presence of nilotinib-resistant UT-7-11 cells. Importantly, this phenomenon was not observed in the screenings involving other TKIs.

**Table 1:** Number and percentage of TKI resistant clones recovered from ENU-treated cells with or without MS-5 feeder and after 5-6 week culture in 96-well plates

Exp.	TKI	UT-7 cell line	Without MS-5 feeder	With MS-5 feeder
1	imatinib	UT-7-11	54 (56%)	51 (53%)
2	nilotinib	UT-7-11	31 (32,3%)	0 (0%)*
3	dasatinib	UT-7-11	12 (12.5%)	11 (11.5%)
4a	ponatinib	UT-7-315	65 (68%)	96 (100%)
4b	ponatinib	UT-7-315	68 (71%)	96 (100%)
5	ponatinib	UT-7-11	0 (0%)	96 (100%)

* No outgrowth observed (viable UT-7-11 located within the MS-5 feeder), 24 wells were replated in methylcellulose in the presence of nilotinib and resistant colonies were observed after 14 days of culture.

Ponatinib experiments were first performed using UT-7-315 cells, which are considered as sensitive to this drug. In the experimental conditions defined above, 68% of the wells were resistant to ponatinib in the absence of MS-5 feeder, whereas all wells appeared resistant to this drug in its presence (Table 1, experiment 4a). These results were reproduced in a second independent experiment (Table 1, experiment 4b). These data clearly suggested that the stromal microenvironment could play a role in resistance to ponatinib, whereas it does not seem to confer an additional resistance to imatinib or dasatinib. In an attempt to differentiate the respective roles of UT-7-315 mutants and MS-5 cell line in ponatinib resistance, mutagenesis assays were also performed starting with UT-7-11 cells (wild-type BCR-ABL1). In the absence of the feeder layer, no growth was observed, whereas all wells displayed outgrowth when cocultured with MS-5, suggesting a specific involvement of the microenvironment in ponatinib resistance (Table 1, experiment 5).

### BCR-ABL1 KD mutations and TKI resistance generated by ENU mutagenesis

The niche-based cell mutagenesis assay performed on UT-7-11 cells or UT-7-315 cells allowed detection of TKI-resistant clones. A series of 24 resistant clones was then randomly selected and tested for BCR-ABL1 KD mutations. Since very few wells appeared positive for dasatinib (12 without MS-5 and 11 with MS-5), all clones were tested. For ponatinib and UT-7-315 cells, 24 clones were picked from each experiment. Concerning nilotinib with MS-5 condition, mutation screening was carried out on hematopoietic colonies after methylcellulose culture. Most of the clones from all experiments were expanded and subsequently analyzed for KD mutations (233/239). As a first step, mutation screening was performed using both DGGE protocol and direct sequencing (Supplementary Fig. S3). Since DGGE did not detect any minority mutant cell populations, direct sequencing was subsequently used alone for all the samples.

In UT-7-11 screening with imatinib and 2^nd^ generation TKIs, only one mutation was found in more than 90% of the clones tested with the exception of the nilotinib selection (Table 2, exp. 1-3). A single clone with a compound mutation was observed in the nilotinib/without MS-5 screening. In addition, no difference was observed according to whether or not the MS-5 feeder was present (imatinib, nilotinib and dasatinib selection). As expected, all mutagenized UT-7-315 cells harbored the T315I mutation. A 2^nd^ mutation was detected in 79% of the cases when MS-5 cells were absent and in only 26% when they were present (Table 2, exp. 4). These data suggest that the stromal niche plays a potential role in ponatinib resistance. The constant detection of T315I mutation in the UT-7-11/ponatinib/with MS-5 screening strengthened this hypothesis (Table 2, exp. 5).

**Table 2:** Resistant clones harboring a BCR-ABL1-KD mutation

Exp.	TKI	UT-7 cell line	MS-5 feeder	Clones tested for mutation		Results of mutation analysis		
				Number	Available**	No mutation	one mutation	two mutations
1	imatinib	UT-7-11	−	24	22	2	20	0
			+	24	23	0	23	0
2	nilotinib	UT-7-11	−	24	24	11	12	1
			+	24*	24	13	11	0
3	dasatinib	UT-7-11	−	12	12	1	11	0
			+	11	11	1	10	0
4	ponatinib	UT-7-315	−	48	47	0	10***	37****
			+	48	46	0	34***	12****
5	ponatinib	UT-7-11	−	0	0	0	0	0
			+	24	24	0	22***	2****

* Methylcellulose colonies after 14 days of culture in the presence of nilotinib.

** Clones correctly expanded in 24-well plates.

*** T315I mutation alone.

**** one mutation in addition to the T315I mutation.

### BCR-ABL1-Kinase Mutation profiles in the absence or in the presence of MS-5 niche

**Figure 1:**
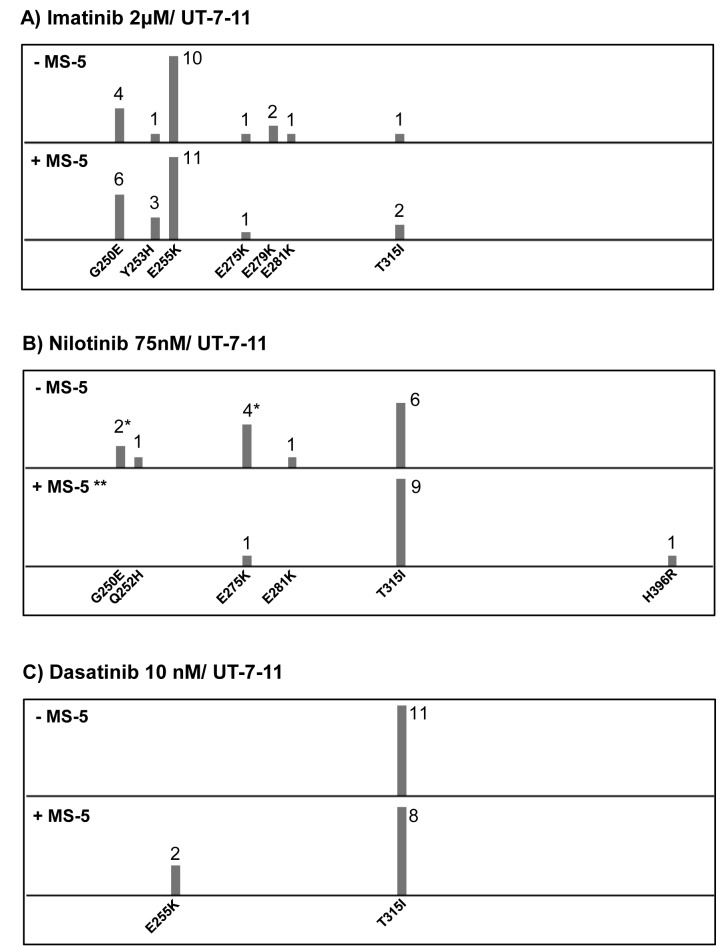
ENU mutagenized resistant clones from UT-7-11 cells treated with 1^st^ and 2^nd^ generation TKIs (imatinib, nilotinib and dasatinib) with or without the stromal cell line MS-5 (A) Clones resistant to imatinib at a concentration of 2μM. (B) Clones resistant to nilotinib at a concentration of 75nM. (C) Clones resistant to dasatinib at a concentration of 10 nM. Each bar represents the number of clones of the indicated KD mutation. * Only one clone carried a compound mutation (G250E+E275K) and was counted in both bars in this condition. ** No outgrowth was observed in this condition; 24 clones were tested after 14 days of culture in methylcellulose.

**Figure 2:**
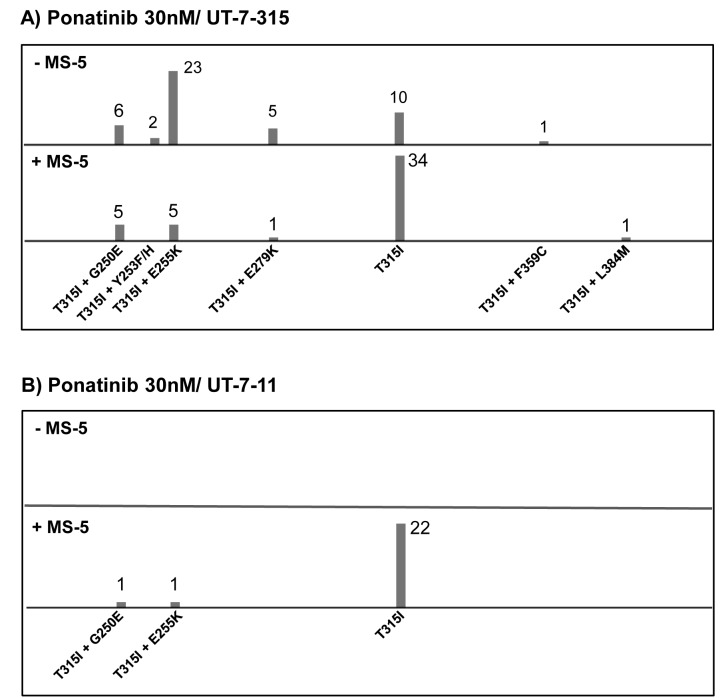
ENU mutagenized resistant clones from UT-7-315 or UT-7-11 cells treated with ponatinib at 30nM with or without the stromal cell line MS-5 (A) UT-7-315 clones resistant to ponatinib at a concentration of 30nM. (B) UT-7-11 clones (native BCR-ABL1) resistant to ponatinib at a concentration of 30nM. Each bar represents the number of clones of the indicated KD mutation or compound mutation.

For 1^st^ and 2^nd^ generation TKIs, the mutation profile appeared almost similar whether or not MS-5 was present. A majority of clones harbored p-loop mutations (G250E, Q252H, Y253H, E255K) or the T315I substitution (Fig. 1). Some other mutations (E275K, E279K, E281K, H396R) were rarely detected. In the nilotinib assay, clones from methylcellulose culture (with MS-5) appeared somewhat enriched in T315I (Fig. 1B). Concerning dasatinib selection, a predominance of the gatekeeper mutation was observed with or without MS-5 feeder (Fig. 1C). Mutation profiles with UT-7-315 and ponatinib selection displayed interesting data. As expected, all resistant clones carried the T315I substitution. In many cases, in addition to the gatekeeper mutation, direct sequencing detected BCR-ABL1 KD mutations located within the p-loop (G250E, Y253F/H, E255K) or elsewhere (E279K, F359C, L384M). When UT-7-315 cells were cultured in the absence of MS-5, clones with compound mutation (T315I + another mutation) represented the majority of resistant clones (Fig. 2A). Conversely, in the presence of MS-5 cells, resistant clones mainly harbored the T315I mutation alone. Consequently, the mutation profiles appeared different according to MS-5 condition. Finally, resistant UT-7-11 cells selected by ponatinib in MS-5 cocultures carried the gatekeeper substitution alone in most cases, whereas no outgrowth was observed without MS-5 (Fig. 2B). Compound mutations were found in only two clones (T315I/G250E, T315I/E255K).

### Array-comparative genomic hybridization experiments

**Figure 3:**
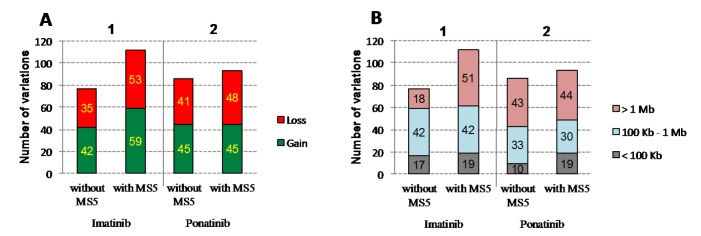
Array CGH analysis of mutant clones obtained from imatinib and ponatinib experiments Potential MS-5 effects were analyzed in (1) two imatinib-resistant UT-7-11 clones harboring the E255K mutation compared with non-mutagenized UT-7-11 control and in (2) two ponatinib-resistant UT-7-315 clones harboring the T315I mutation alone compared with non-mutagenized UT-7-315 control. The total number of variations (losses and gains) is indicated (A) along with variation size (B).

To examine the role of the MS-5 niche, we first analyzed by array-CGH (1) two imatinib-resistant UT-7-11 clones harboring the E255K mutation compared with UT-7-11 control that did not undergo ENU mutagenesis, and (2) two ponatinib-resistant UT-7-315 clones with the T315I mutation alone compared with non-mutagenized UT-7-315 control (Fig. 3). In each condition, one clone was isolated and amplified in the absence of MS-5 stromal cells and the other in the presence of MS-5. Figure 3A reveals the total copy number variations (CNVs) in clones cultured in the presence of MS-5 feeder as compared to clones obtained without MS-5 (112 *vs* 77 for imatinib screening, 93 *vs* 86 for ponatinib screening). In these experiments, size variations are comparable, except for imatinib condition, in which an increase in deletions/insertions >1 Mb was observed with MS-5 (Fig. 3B).

Secondly, direct array-CGH experiments were performed using the culture in the absence of MS-5 as a control, in order to detect potential niche-induced abnormalities. For that purpose, two imatinib-resistant clones harboring E255K and four ponatinib-resistant clones with the E255K/T315I compound mutation (analyzed pairwise) were compared in 3 independent experiments (Supplementary Table S2). A larger number of CNVs were seen in imatinib experiments as compared to ponatinib screening. Interestingly, results from the two independent ponatinib comparisons appeared quite similar. The details of the 3 array-CGH experiments are shown in Supplementary Tables S3-S5. Data pooling allowed us to more precisely examine the recurrent variations (Supplementary Table S6). Generally, five chromosomal loci were identified (1p13.1, 1q42.2, 3q26.31, 9p24.1 and Yq11.223) harboring losses or gains (Supplementary Fig. S4). Using filtering for genes involved in stem cell pluripotency, drug resistance, signal transduction and cancer, a few genes recurrently affected by genomic variations were identified, including *TNFSF10* and *FOXO3* (Table 3).

**Table 3:** Genes involved in stem cell pluripotency, drug resistance, signal transduction or cancer development, recurrently amplified or deleted in the presence of MS-5

Gene	Chromosome location	array-CGH experiment			Full name	Function
		imatinib	ponatinib	ponatinib		
		F3 *vs* E5	A23 *vs* C9	A24 *vs* B4		
CD274	9p24.1	loss	loss	loss	Programmed cell death 1 ligand 1 (PD-L1)	Transmembrane protein, role in immune response
CD58	1p13.1	loss	gain	gain	Lymphocyte function-associated antigen 3 (LFA-3)	Cell molecule adhesion
ECT2	3q26.31	gain	loss	loss	Epithelial cell transforming sequence 2 oncogene	Guanine nucleotide exchange factor, cell communication, signal transduction
TNFSF10	3q26.31	gain	loss	loss	TNF-related apoptosis-inducing ligand (TRAIL)	Receptor binding, cell communication, signal transduction
CDKN2A	9p21.3	(1)	gain	loss	Cyclin-dependent kinase inhibitor 2A (p14ARF)	Cell cycle control protein, cell communication, signal transduction
CCDC6	10q21.2	loss	(1)	loss	Coiled coil domain containing 6	Structural constituent of cytoskeleton, apoptosis
EXT1	8q24.11	(1)	gain	gain	Exostosin glycosyltransferase 1	Glycosyltransferase
FOXO3	6q21	gain	gain	(1)	Forkhead box protein O3A	Transcription factor activity
JAK2	9p24.1	loss	loss	(1)	Janus kinase 2	Protein-tyrosine kinase activity, cell communication, signal transduction
MLLT3	9p21.3	(1)	gain	loss	AF9, Mixed-Lineage Leukemia (Trithorax Homolog, Drosophila) Translocated To 3	Found in acute leukemias with MLL rearrangement
TMPRSS2	21q22.3	loss	loss	(1)	Transmembrane protease serine 2	Serine protease

For each array-CGH experiments, two resistant clones harboring the same mutation were compared (F3 vs E5, A23 vs C9, A24 vs B4). Clones F3, A23 and A24 were recovered from culture with MS-5 feeder. Clones E5, C9 and B4 were recovered from culture without MS-5 feeder. (1) no variation.

## DISCUSSION

Use of TKIs has modified the therapeutic landscape of CML, with a major beneficial effect on overall and disease-free survival. However, a minority of patients develop a resistance to imatinib or 2^nd^ generation TKIs, due in part to BCR-ABL1 kinase domain mutations [17]. This resistance could also be oncogene-independent and mediated by the microenvironment. Recently, several reports have highlighted the involvement of the hematopoietic niche in the leukemogenesis process, leading to new concepts such as “leukemic niche” [18]. In the present work, we used a niche-based cell mutagenesis assay to investigate the role of the hematopoietic niche in resistance towards four drugs used in CML (imatinib, nilotinib, dasatinib and ponatinib). The usefulness of this type of experiment has been demonstrated, in particular for the characterization of TKI resistance profile in CML [11,19]. Moreover, ENU has been shown to be a potent mutagenic agent since it is able to induce point mutations at a rate ~100-fold higher than the rate of spontaneous mutations [20]. In our assay, we used the MS-5 murine mesenchymal stromal cell line, which is known to produce a surrogate hematopoietic niche supporting human hematopoiesis [21]. In addition to this supportive capacity, MS-5 was recently shown to promote vasculogenesis and angiogenesis, two other important processes regulated in and by the niche [22].

In the assay relative to 1^st^ and 2^nd^ generation TKIs, we found that the percentage of UT-7-11 resistant clones decreased from imatinib (more than 50%) to dasatinib (approximately 10%) whatever the MS-5 condition. This percentage appeared intermediate (30%) with nilotinib in the absence of MS-5 feeder. Overall, these results seem to be in accordance with the increasing efficacy of 2^nd^ generation TKIs as reported in the literature [23]. Concerning nilotinib in MS-5 presence, no positive wells were detected. Subsequent microscopic examinations displayed viable cells lodged in the MS-5 niche. However, after replating 24 “negative” wells in methylcellulose, hematopoietic colonies were clearly observed in all cases. With the exception of the latter case, MS-5 stromal niche did not seem to influence resistance towards 1^st^ and 2^nd^ generation TKIs. A BCR-ABL1 KD mutation was characterized in most of the resistant clones recovered from imatinib and dasatinib experiments, and only in half of the cases with nilotinib, regardless of the presence of MS-5 stromal niche. Imatinib/dasatinib mutation profiles did not reveal a significant effect of the MS-5 feeder, but they remained consistent with previous ENU-based mutagenesis studies [19]. P-loop mutations (G250E, Y253H, E255K) and the T315I substitution were detected essentially in the imatinib and dasatinib assays respectively. As previously reported, the nilotinib mutation profile appeared intermediate [24]. In this case, it is uncertain whether or not the MS-5 feeder effects selection of T315I mutants.

Concerning ponatinib assays on UT-7-315 cell line, a sizable increase in the percentage of resistant clones was observed in the presence of MS-5 stromal cells (two independent experiments). When combined with the results of the experiment on UT-7-11 (non-T315I) cells, these data could suggest both the critical role of the surrogate niche, and an environment-mediated resistance phenomenon. Another exciting result was the presence of a majority (78%) of compound mutations (T315I + other mutation) in the absence of MS-5, and the detection of the T315I mutation alone in most of the resistant clones (87.5%) recovered from MS-5 condition. According to these results, we can formulate two hypotheses for T315I-resistance to ponatinib. First, in the absence of the stromal niche, a compound mutation (T315I + another mutation) could be necessary to induce a complete resistance. Indeed, cell-based screenings have demonstrated that ponatinib was potent on single point mutations but less effective on several compound mutations [11]. Second, in the presence of the MS-5 microenvironment, UT-7 cells harboring the T315I mutation alone could survive ponatinib. In this model, T315I mutation and stromal niche could jointly play a crucial role in the acquisition of ponatinib resistance.

T315I substitution is the most frequent BCR-ABL1 KD mutation and leads to complete resistance to 1^st^ and 2^nd^ generation TKIs [17,25]. These T315I mutants appeared to be clearly different from non-mutated BCR-ABL1 leukemic cells. Indeed, T315I mutants have been shown to display an increased oncogenic capability despite reduced tyrosine kinase activity, thereby suggesting altered substrate recognition [26]. The gatekeeper mutation has also been associated with severe transformation potency in the absence of fetal bovine serum or growth factors [27]. In addition, it has been suggested that T315I mutation may confer additional leukemogenic activity to non-mutated BCR-ABL1 related to the phosphorylation of endogenous BCR [28]. All of these features could give rise to T315I “specific signaling pathways”, highlighting the gatekeeper substitution as the most particular BCR-ABL1 KD mutation. These gain-of-function properties could therefore be associated with extrinsic mechanisms of resistance. However, ponatinib has been shown to be active against BCR-ABL1 mutants (in particular the T315I mutant) *in vitro* as well as *in vivo* [11,29]. Altered BCR-ABL1 signaling potentially due to the T315I mutation, associated with a stroma-mediated influence on these particular signaling pathways, could represent an explanation for ponatinib escape. Based on IC50 values, it is noticeable that T315I show an intermediate resistance to ponatinib [30]. Moreover, it has recently been reported that TKI IC50 values were substantially increased in the presence of human stromal cell conditioned media [31]. Overall, these data suggest that T315I mutants, supported by a stromal niche, could survive ponatinib through interactions mediated by direct contact or soluble factors.

Regarding the potential support to TKI resistance provided by MS-5 stromal cells, we wondered whether these cells could promote genomic instability. It has already been reported that this phenomenon could be induced by tumoral microenvironment, resulting in genomic events influencing drug resistance [32]. Critical components regulated by the niche as hypoxia or metalloproteinases have been shown to play a sizable role in genomic instability and in the production of reactive oxygen species [33,34]. In the present study, array-CGH experiments suggest a slight involvement of MS-5 niche in the number of genetic modifications. Among the recurring abnormalities, the gain of genomic regions including the *FOXO3* gene can be of interest. This transcription factor was reported to be crucial to the maintenance of leukemic cells in CML via TGF-β signaling [35]. The *TNFSF10* (TRAIL) gene, located in the 3q26.31 region gained in imatinib or lost in ponatinib experiments, has been shown to sensitize imatinib-resistant cells to apoptosis by down-regulating *c-FLIP*. This latter gene is implicated in oncogene-independent resistance on account of the fact that leukemic cells exhibit attenuated BCR-ABL1 activity [36]. Since it has been suggested that the hematopoietic niche could induce a TRAIL-mediated apoptosis, investigation of this pathway in the context of CML resistance (especially with ponatinib) could be of importance [37,38]. Finally, the *CDKN2A/B* locus appears to be involved in the progression of CML, and JAK2 could play a role in TKI resistance through a BCR-ABL1/JAK2 network [39,40].

Nowadays, in clinical practice, the influence of BCR-ABL1 KD mutations in TKI resistance is decreasing. Causes of mutation-independent resistance, particularly the role of the hematopoietic niche, are currently under investigation. Primitive HSCs are refractory to all of the TKIs used in clinical practice [12-14]. This resistance can be due not only to the persistence of quiescent leukemic cells, but also to molecular mechanisms of resistance related to the bone marrow microenvironment [41,42]. The hematopoietic niche could then appear as a critical target to cure CML [43]. Stroma-mediated protection from TKI treatment has in fact been reported in several works, all of which highlight the critical role of the hematopoietic niche in TKI resistance [44,45].

In conclusion, we have reported a niche-based cell mutagenesis assay performed on human UT-7 cells that allowed for screening of TKI-resistant BCR-ABL1-expressing clones. For the first time, we have described stromal-mediated ponatinib resistance for BCR-ABL1^T315I^ mutants. In the absence of MS-5 niche, compound mutations appeared to be the major way to escape ponatinib therapy, especially via the occurrence of p-loop mutations in addition to T315I. In the presence of the niche, resistance appeared due to the close association of T315I mutants and stromal cells, suggesting a cross-talk between the two components. Overall, we conclude that stromal niche could play a crucial role in conferring resistance to ponatinib, by providing survival signals and favoring genetic instability, which is likely to lead to the occurrence of novel mutations.

## MATERIALS AND METHODS

### Cell lines

In the present assay, the murine stromal cell line MS-5 was used as a niche model. UT-7 cell line, established from a patient with acute megakaryoblastic leukemia, was used as a model of leukemic cells [46]. UT-7-11 cells were generated by transduction of native BCR-ABL1 gene as previously described [47]. UT-7 cells harboring the T315I BCR-ABL1 (UT-7-315) were generated by lipofectamine transfection of a MIGR-BCR-ABL1-T315I plasmid.

### Drugs and reagents

TKIs (imatinib, nilotinib, dasatinib and ponatinib) used in this study were purchased from Selleck Chemicals (Houston, Texas) and stored at -20 °C as 1mM stock solutions in DMSO. Before experiments, fresh dilutions were prepared with culture medium. ENU (Sigma Aldrich, St. Louis, Missouri) was reconstituted with DMSO as 50 mg/ml stock solution, aliquoted and stored at -80°C.

### In Vitro-ENU Mutagenesis

UT-7-11 or UT-7-315 were cultured in complete MEM alpha medium (Life Technologies, Carlsbad, California) containing 10% FBS, L-glutamine (Life Technologies) and 1% penicillin/streptomycin (Life Technologies). ENU was added to UT-7 cells at a concentration of 50 μg/ml, followed by a 24h culture. This dose was minimally toxic to our cells (20% cell death). Cells were then washed three times with culture medium, replated in complete medium and expanded over one week.

### Resistance screening and mutant cell expansion

ENU exposed UT-7 cells were cultured in 96-well plates at 10^5^ cells/well in 200 μl medium in the presence or in the absence of MS-5 and in the presence of either imatinib (2μM), nilotinib (75nM), dasatinib (10nM) or ponatinib (30nM). MS-5 cells were plated a day before in 96-well plates at a concentration of 6000 cells/well. Wells were monitored for cell growth by microscopic inspection twice a week for 5-6 weeks. 24 positive wells, randomly picked, were then transferred to 24-well plates and expanded using the same concentration of TKI (Supplementary Fig. S1).

### Detection of BCR-ABL1 KD mutations

After expansion, TKI-resistant cells were lyzed in Trizol solution (Trizol reagent, Life Technologies). Total RNA was extracted using manufacturer's protocol, and cDNA was prepared from 2μg of total RNA using the High-Capacity cDNA Reverse transcription kit (Life Technologies). The BCR-ABL1 tyrosine kinase domain (corresponding to amino acids 242 to 427) was amplified by two overlapping PCR reactions and DGGE experiments were performed as previously described [48]. For direct sequencing, PCR products were purified (Illustra Exostar 1-step, VWR, Radnor, Pennsylvania) and sequenced using the BigDye Terminator v3.1 Cycle sequencing kit on an ABI 3500Dx (Life Technologies). Mutation analysis was carried out using the Mutation Surveyor software (SoftGenetics, State College, PA).

### Oligonucleotide array-comparative genomic hybridization (array-CGH)

Array-CGH was conducted on UT-7-11 and UT-7-315 mutant clones selected from imatinib and ponatinib screenings respectively. As a control, we used UT-7 cell lines that did not undergo ENU mutagenesis. Genomic DNA was isolated using DNeasy Blood and Tissu Kit (Qiagen, Courtaboeuf, France). DNA concentrations were estimated using a NanoDrop ND-1000 spectrophotometer (NanoDrop Technologies, Wilmington, DE). The genomic imbalances were analyzed using the human CGH 12x135K Whole-Genome Tiling v3.0 Array (Roche NimbleGen, Meylan, France). All array hybridizations were performed according to the manufacturer's recommended protocols. Briefly, 0.5 μg of genomic DNA were fluorescently labeled with the Roche NimbleGen Dual-Color DNA labeling kit (Roche NimbleGen). Male human genomic DNA (Promega, Charbonnière, France) was used as reference. Cy5-dUTP cell line DNA and gender-matched reference DNA labeled with Cy3-dUTP were denatured prior to hybridization (NimbleGen Hybridization kit) for 48 h at 42°C in the Hybridization System 4 (Roche NimbleGen). After washing (NimbleGen Wash Buffer Kit), the slides were scanned on a Roche NimbleGen MS200 Microarray Scanner. Captured images were processed with NimbleScan software and data analysis was performed with DEVA software v1.0.2 (Roche Nimblegen). The Nexus Copy Number Standard edition software (Proteigene, Saint-Marcel, France) algorithm was used for statistical analysis according to the version 18 of the Human genome built (http://genome.ucsc.edu/). Copy number variations (CNV) were considered significant if they were defined by 5 or more oligonucleotides spanning at least 50 Kb.

## SUPPLEMENTARY FIGURES AND TABLES


