# Supplementary material for: Modeling the influence of stromal microenvironment in the selection of ENU-induced BCR-ABL1 mutants by tyrosine kinase inhibitors
Source: Oncoscience. 2014 Jan 30;1(1):57–68. doi: 10.18632/oncoscience.9 (PMC4295758; doi:10.18632/oncoscience.9)
Supplement: Supplementary file 1 [file oncoscience-01-0057-s001.pdf]

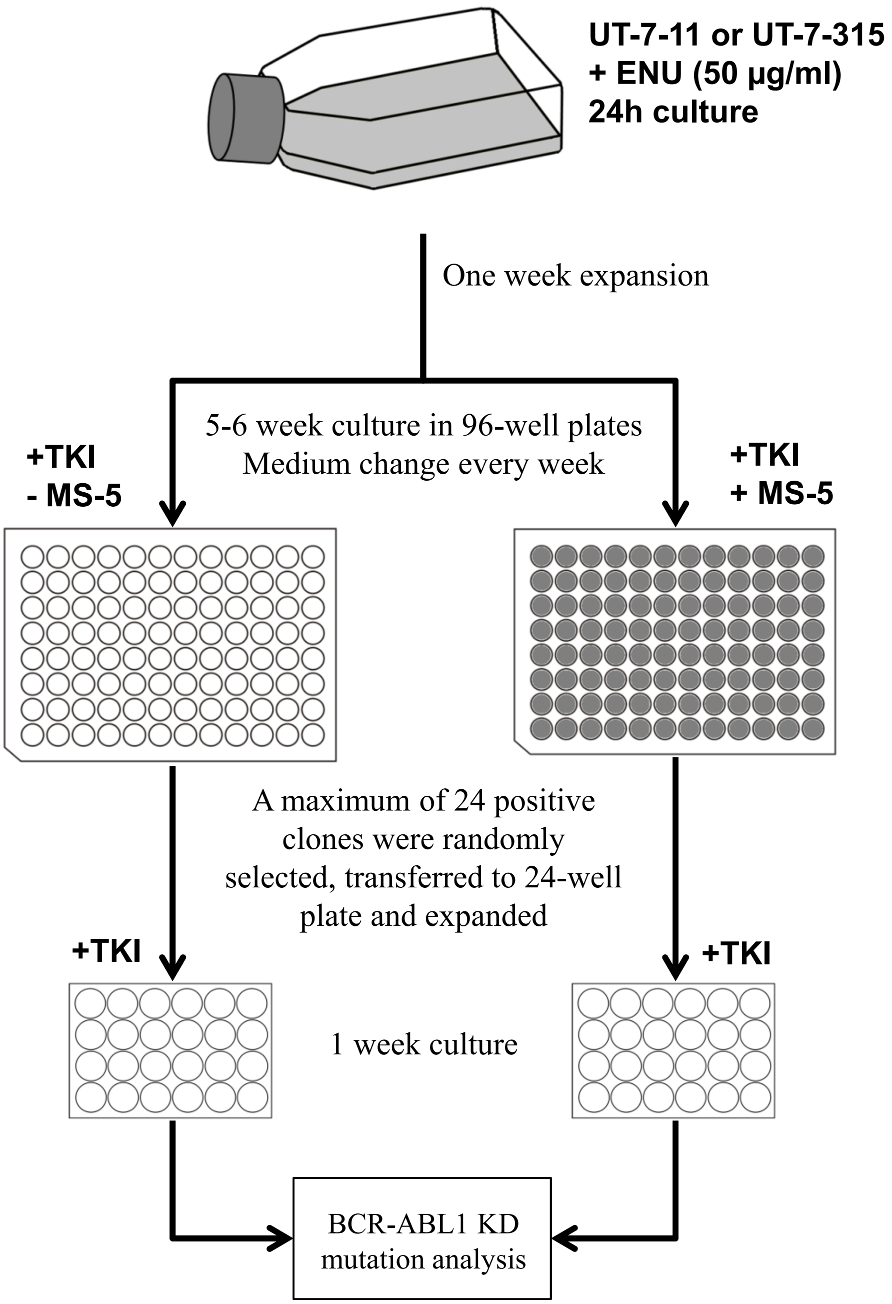


**Fig. S1. Design of the mutagenesis assay.**

UT-7 cell lines expressing native or T315I-mutated BCR-ABL1 were used. Mutagenesis was carried out with N-ethyl-N-Nitrosourea (ENU) for 24h in culture at a concentration of 50 µg/ml. Cells were then washed three times with culture medium, replated in complete medium and expanded over one week. ENU exposed UT-7 cells were then cultured in 96-well plates at $10^5$cells/well in 200 µl medium in the presence or in the absence of MS-5 feeder. They were cultured with the following TKI concentrations: imatinib 2µM, nilotinib 75nM, dasatinib 10nM, ponatinib 30nM. When used as a niche model, MS-5 cells were plated a day before in the 96-well plates at a concentration of 6000 cells/well. Cell growth was monitored by microscopic inspection twice a week for 5-6 weeks. A maximum of 24 positive wells (randomly chosen) were then transferred to 24-well plates and expanded using the same TKI concentration for subsequent BCR-ABL1 kinase domain (KD) mutation analysis.

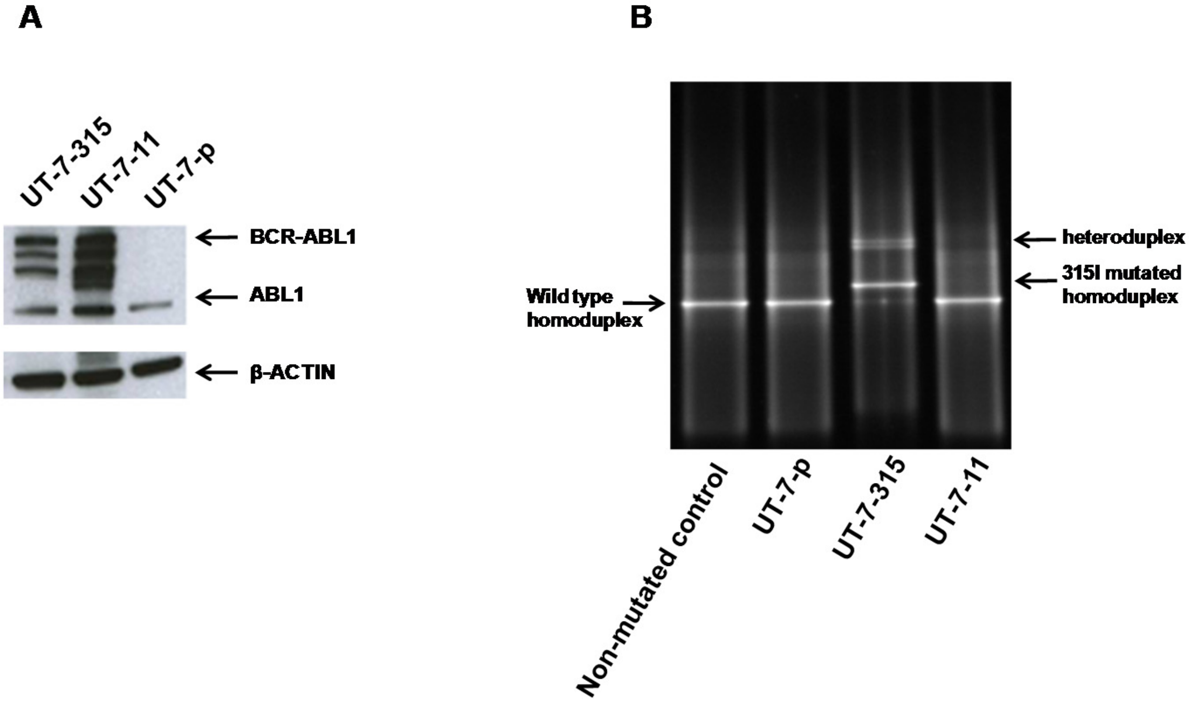


**Fig. S2. Characterization of UT-7 cell lines.**
(A) Western-blot analysis showed that the BCR-ABL1 oncoprotein is present in UT-7-11 and UT-7-315, but not in UT-7 parental (UT-7-p) cell line from which they derive. (B) Mutation screening of the BCR-ABL1 kinase domain by DGGE (Denaturing Gradient Gel Electrophoresis) shows the presence of the gatekeeper mutation in UT-7-315 cell line and confirms the absence of any mutation in UT-7 parental or UT-7-11 cell lines as compared to non-mutated control.

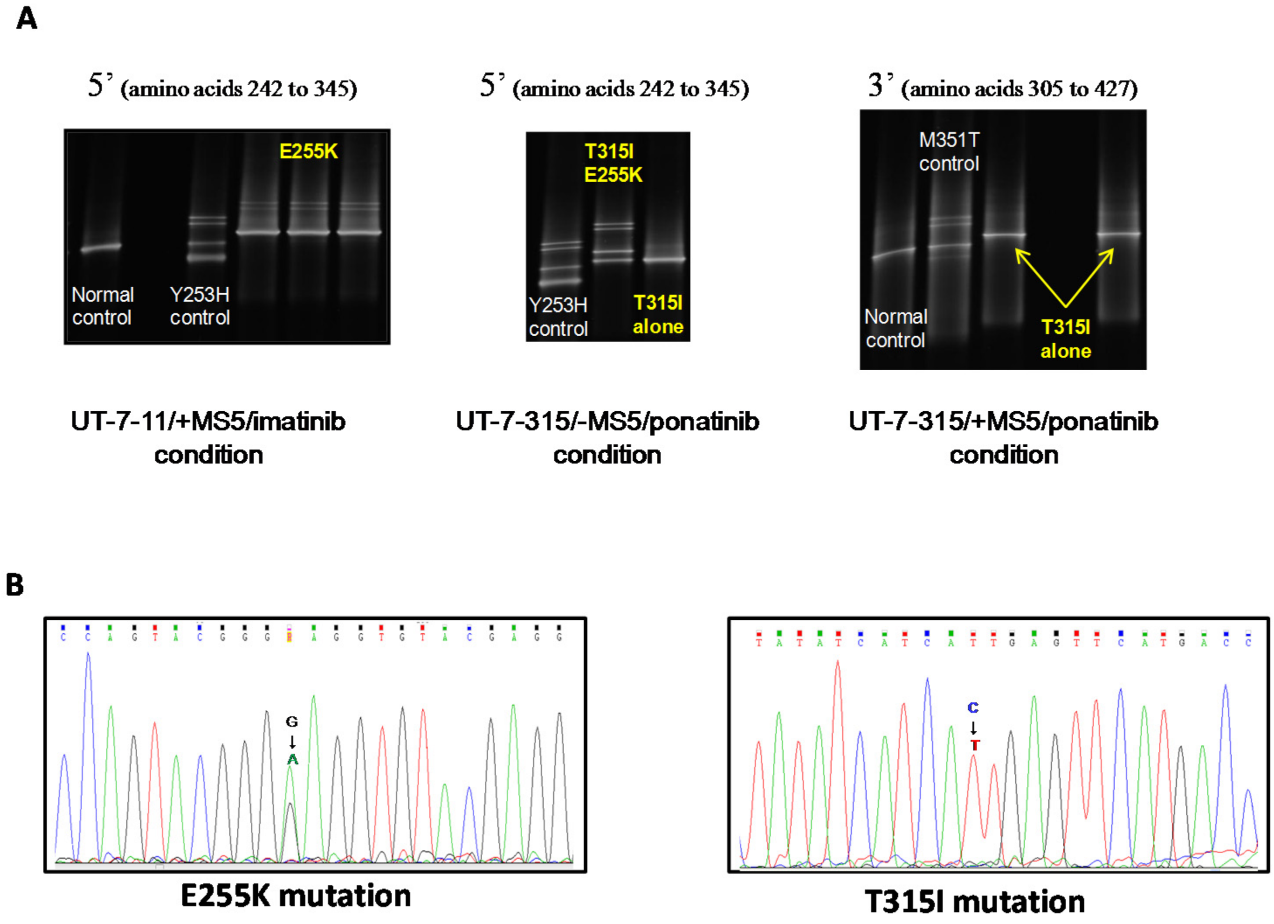


**Fig. S3: Examples of BCR-ABL1-KD mutation screening.**
(A) DGGE screening of the 5' end (amino acids 242 to 345) or the 3' end (amino acids 305 to 427) of the BCR-ABL1 kinase domain. (B) Direct sequencing characterization of E255K and T315I mutations.

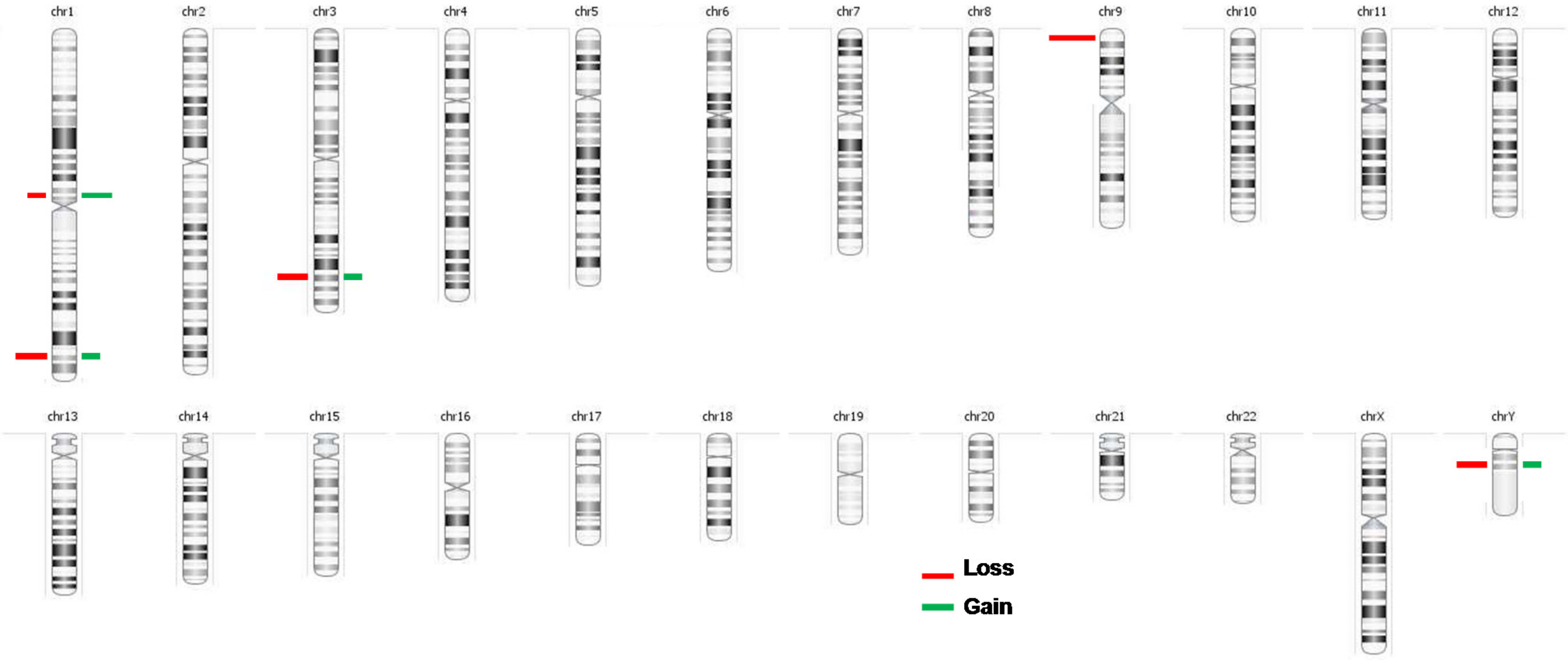


**Fig. S4: Recurrent chromosome variations observed in imatinib-resistant UT-7-11 cells (n=1) and ponatinib-resistant UT-7-315 cells (n=2) according to MS-5 presence.**

**Table S1:** UT-7 cell mortality determined at the plateau concentration for each TKI (n=3).

| UT-7 cell lines | | no TKI | imatinib (1µM) | nilotinib (50nM) | dasatinib (5nM) | ponatinib (15nM) |
|---|---|---|---|---|---|---|
| **UT-7-p** | | 12% | 14.4% | 16.7% | 21% | 21% |
| **UT-7-11** | wild type BCR-ABL1 | 16.2% | **80%** | **86%** | **86%** | **91.5%** |
| **UT-7-315** | T315I-mutated BCR-ABL1 | 9% | 9% | 7% | 17% | **86%** |

UT-7-p : UT-7 parental.

**Table S2:** Comparison of resistant clones cultured in the presence or in the absence of MS-5 feeder by array-CGH.

| **Cell line/ TKI** | **With MS-5 feeder** | **Without MS-5 feeder (used as calibrator)** | **Total number of variations** | **Losses** | **Gains** |
|---|---|---|---|---|---|
| UT-7-11 imatinib | clone F3 *E255K* | clone E5 *E255K* | 117 | 57 | 60 |
| UT-7-315 ponatinib | clone A23 *E255K + T315I* | clone C9 *E255K + T315I* | 58 | 31 | 27 |
| UT-7-315 ponatinib | clone A24 *E255K + T315I* | clone B4 *E255K + T315I* | 59 | 32 | 27 |

Mutation(s) detected in UT-7 clones are in italic.

**Table S3.** Array-CGH analysis comparing imatinib-resistant UT-7-11 clones F3 (with MS-5) *vs* E5 (without MS-5).

| Chromosome Region (hg18) | Event | Length (Kb) | Cytoband | Genes | miRNAs | Cancer Genes (Cancer Gene Census) |
|---|---|---|---|---|---|---|
| chr1:52,174,291-53,101,776 | Gain | 927,486 | p32.3 | BTF3L4, CC2D1B, FAM159A, GPX7, KTI12, ORC1, PRPF38A, RAB3B, SELRC1, TXNDC12, ZCCHC11, ZFYVE9, ZYG11A, ZYG11B | | |
| chr1:116,337,471-116,919,312 | Loss | 581,842 | p13.1 | ATP1A1, ATP1A1OS, CD58, IGSF3, MAB21L3, SLC22A15 | | |
| chr1:117,306,259-118,883,465 | Gain | 1577,207 | p13.1 - p12 | CD101, FAM46C, GDAP2, MAN1A2, MIR942, PTGFRN, SPAG17, TRIM45, TTF2, VTCN1, WDR3 | hsa-mir-942 | |
| chr1:148,467,954-149,948,427 | Gain | 1480,474 | q21.2 - q21.3 | ADAMTSL4, ANP32E, ANXA9, APH1A, ARNT, BNIPL, C1orf51, C1orf54, C1orf56, CA14, CDC42SE1, CELF3, CERS2, CGN, CTSK, CTSS, ECM1, ENSA, FAM63A, GABPB2, GOLPH3L, HORMAD1, LINC00568, LYSMD1, MCL1, MIR4257, MIR554, MLLT11, MRPS21, PI4KB, PIP5K1A, POGZ, PRPF3, PRUNE, PSMB4, PSMD4, RFX5, RPRD2, SCNM1, SELENBP1, SEMA6C, SETDB1, SNX27, TARS2, TMOD4, TNFAIP8L2, TNFAIP8L2-SCNM1, TUFT1, VPS72, ZNF687 | hsa-mir-554 | ARNT, MLLT11 |
| chr1:151,670,991-154,822,634 | Gain | 3151,644 | q21.3 - q23.1 | ADAM15, ADAR, AQP10, ARHGEF2, ASH1L, ASH1L-AS1, ATP8B2, BGLAP, C1orf189, C1orf43, C1orf61, C1orf85, CCT3, CHRNB2, CHTOP, CKS1B, CLK2, CREB3L4, CRTC2, DAP3, DCST1, DCST2, DENND4B, DPM3, EFNA1, EFNA3, EFNA4, FAM189B, FDPS, FLAD1, GATAD2B, GBA, GBAP1, GON4L, HAX1, HCN3, IL6R, ILF2, INTS3, IQGAP3, JTB, KCNN3, KIAA0907, KRTCAP2, LAMTOR2, LENEP, LMNA, LOC100505666, MEF2D, MEX3A, MIR190B, MIR4258, MIR555, MIR9-1, MIR92B, MSTO1, MSTO2P, MTX1, MUC1, NPR1, NUP210L, PAQR6, PBXIP1, PKLR, PMF1, PMF1-BGLAP, PMVK, POU5F1P4, PYGO2, RAB13, RAB25, RHBG, RIT1, RPS27, RUSC1, RUSC1-AS1, RXFP4, S100A1, S100A13, S100A14, S100A16, S100A2, S100A3, S100A4, S100A5, S100A6, S100A7, S100A7L2, SCAMP3, SCARNA4, SEMA4A, SHC1, SHE, SLC25A44, SLC27A3, SLC39A1, SLC50A1, SMG5, SNAPIN, SNORA42, SSR2, SYT11, TDRD10, THBS3, TMEM79, TPM3, TRIM46, TSACC, TTC24, UBAP2L, UBE2Q1, UBQLN4, VHLL, YY1AP1, ZBTB7B | hsa-mir-9-1, hsa-mir-190b, hsa-mir-92b, hsa-mir-555 | TPM3, MUC1 |
| chr1:159,754,964-159,951,019 | Gain | 196,056 | q23.3 | FCGR2A, FCGR2B, FCGR2C, FCGR3A, FCGR3B, FCRLA, HSPA6, HSPA7, RPL31P11 | | FCGR2B |
| chr1:200,669,183-204,599,252 | Gain | 3930,07 | q32.1 | ADIPOR1, ADORA1, ATP2B4, AVPR1B, BTG2, C1orf186, CDK18, CHI3L1, CHIT1, CNTN2, CTSE, CYB5R1, DSTYK, ELK4, ETNK2, FAM72A, FMOD, GOLT1A, KDM5B, KDM5B-AS1, KISS1, KLHDC8A, KLHL12, LAX1, LEMD1, LEMD1-AS1, LINC00260, LINC00303, LINC00628, LOC148709, LOC284578, LOC284581, LOC401980, LOC641515, LOC730227, LRRN2, MDM4, MFSD4, MIR135B, MYBPH, MYOG, NFASC, NUAK2, NUCKS1, OPTC, PIK3C2B, PLEKHA6, PM20D1, PPFIA4, PPP1R12B, PPP1R15B, PRELP, RAB7L1, RABIF, RBBP5, REN, SLC26A9, SLC41A1, SLC45A3, SNORA77, SNRPE, SOX13, SRGAP2, SYT2, TMCC2, TMEM183A, TMEM183B, TMEM81, ZBED6, ZC3H11A | hsa-mir-135b | MDM4, ELK4, SLC45A3 |

| Chromosome Region (hg18) | Event | Length (Kb) | Cytoband | Genes | miRNAs | Cancer Genes (Cancer Gene Census) |
|---|---|---|---|---|---|---|
| chr1:206,192,979-209,374,590 | Loss | 3181,612 | q32.2 | C1orf74, CAMK1G, DIEXF, G0S2, HHAT, HSD11B1, IRF6, KCNH1, LAMB3, MIR205, MIR205HG, MIR4260, PLXNA2, SERTAD4, SERTAD4-AS1, SYT14, TRAF3IP3 | hsa-mir-205 | |
| chr1:211,536,743-229,774,281 | Loss | 18237,54 | q32.3 - q42.2 | ABCB10, ACBD3, ACTA1, ADCK3, AGT, AIDA, ARF1, ARV1, AURKAPS1, BPNT1, BROX, C1orf115, C1orf131, C1orf140, C1orf145, C1orf198, C1orf35, C1orf65, C1orf95, CAPN2, CAPN8, CAPN9, CCSAP, CDC42BPA, CENPF, CNIH3, CNIH4, COG2, DEGS1, DISP1, DNAH14, DUSP10, DUSP5P1, EGLN1, ENAH, EPHX1, EPRS, ESRRG, EXOC8, FAM177B, FAM89A, FBXO28, GALNT2, GJC2, GNPAT, GPATCH2, GUK1, H3F3A, H3F3AP4, HHIPL2, HIST3H2A, HIST3H2BB, HIST3H3, HLX, IARS2, IBA57, ITPKB, JMJD4, KCNK2, KCTD3, LBR, LEFTY1, LEFTY2, LIN9, LINC00210, LINC00538, LOC100130093, LOC149373, LOC643723, LOC728463, LYPLAL1, MARC1, MARC2, MARK1, MIA3, MIR1182, MIR194-1, MIR215, MIR320B2, MIR3620, MIR4666A, MIR4742, MIR5008, MIR548F3, MIR664, MIXL1, MRPL55, NUP133, NVL, OBSCN, PARP1, PGBD5, PROX1, PRSS38, PSEN2, PTPN14, PYCR2, RAB3GAP2, RAB4A, RHOU, RNF187, RNU5F-1, RRP15, SDE2, SLC30A10, SMYD2, SNAP47, SNORA36B, SNRPD2P2, SPATA17, SPHAR, SPRTN, SRP9, SUSD4, TAF1A, TAF5L, TGFB2, TLR5, TMEM63A, TP53BP2, TRIM11, TRIM17, TRIM67, TSNAX, TSNAX-DISC1, TTC13, URB2, USH2A, WDR26, WNT3A, WNT9A, ZNF678, ZNF847P | hsa-mir-1182, hsa-mir-320b-2, hsa-mir-664, hsa-mir-194-1, hsa-mir-215 | |
| chr2:45,857-8,992,327 | Loss | 8946,471 | p25.3 - p25.1 | ACP1, ADI1, ALLC, CMPK2, COLEC11, FAM150B, ID2, KIDINS220, LINC00299, LINC00487, LOC100505964, LOC100506054, LOC100506274, LOC150622, LOC339788, LOC339822, LOC400940, LOC727982, LOC730811, MBOAT2, MYT1L, PXDN, RNASEH1, RNF144A, RNF144A-AS1, RPS7, RSAD2, SH3YL1, SNTG2, SOX11, TMEM18, TPO, TRAPPC12, TSSC1 | | |
| chr2:11,458,171-23,776,383 | Loss | 12318,21 | p25.1 - p24.1 | APOB, C2orf43, DDX1, E2F6, FAM49A, FAM84A, FLJ12334, GDF7, GEN1, GREB1, HS1BP3, KCNS3, KLHL29, LAPTM4A, LINC00570, LOC100506474, LOC645949, LPIN1, MATN3, MIR3125, MIR3681, MIR4262, MIR4429, MIR4757, MSGN1, MYCN, MYCNOS, NBAS, NT5C1B, NT5C1B-RDH14, NTSR2, OSR1, PUM2, RAD51AP2, RDH14, RHOB, SDC1, SMC6, TRIB2, TTC32, VSNL1, WDR35 | | MYCN |
| chr2:29,298,600-31,772,887 | Loss | 2474,288 | p23.2 - p23.1 | ALK, CAPN13, CAPN14, EHD3, GALNT14, LBH, LCLAT1, SRD5A2, XDH, YPEL5 | | ALK |
| chr2:100,429,953-100,462,718 | Gain | 32,766 | q11.2 | NMS | | |
| chr2:107,628,543-108,638,831 | Gain | 1010,289 | q12.3 - q13 | GCC2, LIMS1, LOC729121, RGPD4, SLC5A7, SULT1C2, SULT1C2P1, SULT1C3, SULT1C4 | | |
| chr2:113,890,983-114,010,857 | Loss | 119,875 | q14.1 | CBWD2, FOXD4L1 | | |
| chr2:144,711,391-144,860,090 | Gain | 148,7 | q22.3 | GTDC1, ZEB2 | | |
| chr2:148,771,371-149,115,036 | Gain | 343,666 | q23.1 | MBD5 | | |

| Chromosome Region (hg18) | Event | Length (Kb) | Cytoband | Genes | miRNAs | Cancer Genes (Cancer Gene Census) |
|---|---|---|---|---|---|---|
| chr2:183,303,714-183,585,338 | Gain | 281,625 | q32.1 | DNAJC10, FRZB, NCKAP1 | | |
| chr2:187,041,361-188,127,412 | Gain | 1086,052 | q32.1 | CALCRL, FAM171B, ITGAV, TFPI, ZC3H15, ZSWIM2 | | |
| chr2:201,539,011-204,172,649 | Gain | 2633,639 | q33.1 - q33.2 | ABI2, ALS2, ALS2CR11, ALS2CR12, ALS2CR8, BMPR2, CASP10, CASP8, CDK15, CFLAR, CFLAR-AS1, CYP20A1, FAM117B, FAM126B, FZD7, ICA1L, MPP4, NBEAL1, NDUFB3, NOP58, RAPH1, SNORD11, SNORD11B, SNORD70, STRADB, SUMO1, TMEM237, TRAK2, WDR12 | | |
| chr2:241,833,989-242,460,220 | Gain | 626,232 | q37.3 | ATG4B, BOK, BOK-AS1, D2HGDH, DTYMK, FARP2, GAL3ST2, HDLBP, ING5, NEU4, PDCD1, SEPT2, STK25, THAP4 | | |
| chr3:49,820-3,746,746 | Loss | 3696,927 | p26.3 - p26.2 | CHL1, CNTN4, CNTN4-AS2, CNTN6, CRBN, IL5RA, TRNT1 | | |
| chr3:4,299,772-4,674,599 | Loss | 374,828 | p26.2 | ITPR1, SETMAR, SUMF1 | | |
| chr3:4,801,049-9,224,242 | Loss | 4423,194 | p26.2 - p25.3 | ARL8B, BHLHE40, BHLHE40-AS1, CAV3, EDEM1, GRM7, ITPR1, LINC00312, LMCD1, LMCD1-AS1, MIR4790, OXTR, RAD18, SRGAP3, SSUH2 | | SRGAP3 |
| chr3:10,346,128-19,752,961 | Loss | 9406,834 | p25.3 - p24.3 | ANKRD28, ATG7, ATP2B2, BTD, C3orf20, CAND2, CAPN7, CCDC174, CHCHD4, COL6A4P1, COLQ, DAZL, DPH3, EAF1, FBLN2, FGD5, FGD5-AS1, FGD5P1, GALNTL2, GRIP2, HACL1, HDAC11, HRH1, IQSEC1, KCNH8, LINC00606, LINC00620, LOC100129480, LOC339862, LSM3, METTL6, MIR3714, MIR4270, MIR4791, MIR563, MIR885, MKRN2, MRPS25, NR2C2, NUP210, OXNAD1, PLCL2, PPARG, RAF1, RFTN1, RPL32, SATB1, SH3BP5, SH3BP5-AS1, SLC6A1, SLC6A1-AS1, SLC6A11, SLC6A6, SNORA7A, SYN2, TAMM41, TBC1D5, TIMP4, TMEM40, TMEM43, TPRXL, TSEN2, VGLL4, WNT7A, XPC, ZFYVE20 | hsa-mir-885, hsa-mir-563 | PPARG, RAF1, XPC |
| chr3:20,727,572-31,336,733 | Loss | 10609,16 | p24.3 - p23 | AZI2, CMC1, EOMES, GADL1, LOC152024, LOC285326, LOC644990, LOC645206, LRRC3B, MIR4442, MIR4792, NEK10, NGLY1, NKIRAS1, NR1D2, OXSM, RARB, RBMS3, RPL15, SLC4A7, TGFBR2, THRB, TOP2B, UBE2E1, UBE2E2, VENTXP7, ZCWPW2, ZNF385D | | |
| chr3:33,920,498-38,853,588 | Loss | 4933,091 | p22.3 - p22.2 | ACAA1, ACVR2B, ACVR2B-AS1, ARPP21, C3orf35, CTDSPL, DCLK3, DLEC1, EPM2AIP1, EXOG, GOLGA4, ITGA9, LRRFIP2, MIR128-2, MIR26A1, MLH1, MYD88, OXSR1, PLCD1, SCN10A, SCN5A, SLC22A13, SLC22A14, STAC, TRANK1, VILL, XYLB | hsa-mir-26a-1, hsa-mir-128-2 | MLH1, MYD88 |
| chr3:53,584,702-56,496,815 | Loss | 2912,114 | p21.1 - p14.3 | ACTR8, CACNA1D, CACNA2D3, CACNA2D3-AS1, CHDH, ERC2, ERC2-IT1, ESRG, IL17RB, LRTM1, MIR3938, SELK, WNT5A | | |
| chr3:58,943,743-60,620,802 | Loss | 1677,06 | p14.2 | C3orf67, FHIT | | FHIT |
| chr3:60,960,937-61,101,375 | Loss | 140,439 | p14.2 | FHIT | | FHIT |
| chr3:62,685,505-68,801,741 | Loss | 6116,237 | p14.2 - p14.1 | ADAMTS9, ADAMTS9-AS2, ATXN7, C3orf49, CADPS, FAM19A1, KBTBD8, LOC100287879, LOC285401, LRIG1, MAGI1, MAGI1-AS1, MIR4272, MIR548AN, PRICKLE2, PRICKLE2-AS1, PRICKLE2-AS2, PRICKLE2-AS3, PSMD6, PSMD6-AS2, SLC25A26, SNTN, SUCLG2, SYNPR, SYNPR-AS1, THOC7 | | |

| Chromosome Region (hg18) | Event | Length (Kb) | Cytoband | Genes | miRNAs | Cancer Genes (Cancer Gene Census) |
|---|---|---|---|---|---|---|
| chr3:73,423,925-87,858,993 | Loss | 14435,07 | p13 - p11.2 | CADM2, CADM2-AS2, CHMP2B, CNTN3, FAM86DP, FLJ20518, FRG2C, GBE1, LOC401074, LOC440970, MIR1324, MIR4273, MIR4444-1, MIR4795, MIR5688, PDZRN3, POU1F1, RNU6-69, ROBO1, ROBO2, VGLL3, ZNF717 | hsa-mir-1324 | |
| chr3:173,711,933-174,194,891 | Gain | 482,959 | q26.31 | ECT2, NCEH1, SPATA16, TNFSF10 | | |
| chr3:180,071,299-180,846,962 | Gain | 775,664 | q26.32 - q26.33 | ACTL6A, GNB4, KCNMB3, MFN1, MRPL47, NDUFB5, PIK3CA, ZMAT3, ZNF639 | | PIK3CA |
| chr5:31,570,156-32,645,451 | Gain | 1075,296 | p13.3 | C5orf22, GOLPH3, MIR4279, MTMR12, PDZD2, SUB1, ZFR | hsa-mir-579 | |
| chr5:56,675,009-59,878,074 | Loss | 3203,066 | q11.2 - q12.1 | ACTBL2, GAPT, PART1, PDE4D, PLK2, RAB3C | hsa-mir-582 | |
| chr6:34,768,585-34,876,380 | Gain | 107,796 | p21.31 | C6orf106, SNRPC, UHRF1BP1 | | |
| chr6:107,645,048-111,458,933 | Gain | 3813,886 | q21 | AKD1, AMD1, ARMC2, CCDC162P, CD164, CDC40, CDK19, CEP57L1, DDO, FIG4, FOXO3, GPR6, GTF3C6, LACE1, LINC00222, METTL24, MICAL1, NR2E1, OSTM1, PDSS2, PPIL6, RPF2, SCML4, SEC63, SESN1, SLC22A16, SMPD2, SNX3, SOBP, WASF1, ZBTB24 | | FOXO3 |
| chr6:135,284,717-135,377,461 | Gain | 92,745 | q23.3 | ALDH8A1, HBS1L, MIR3662 | | |
| chr6:135,377,520-135,539,456 | Gain | 161,937 | q23.3 | HBS1L | | |
| chr6:151,939,874-158,834,033 | Gain | 6894,16 | q25.1 - q25.3 | ARID1B, CCDC170, CLDN20, CNKSR3, ESR1, FBXO5, GTF2H5, IPCEF1, MIR3692, MIR4466, MTRF1L, MYCT1, NOX3, OPRM1, RGS17, SCAF8, SERAC1, SNX9, SYNE1, SYNJ2, SYNJ2-IT1, TFB1M, TIAM2, TULP4, VIP, ZDHHC14 | hsa-mir-1202 | |
| chr7:38,515,933-39,557,127 | Loss | 1041,195 | p14.1 | AMPH, FAM183B, POU6F2, POU6F2-AS1, VPS41 | | |
| chr7:40,808,686-42,997,611 | Loss | 2188,926 | p14.1 | C7orf10, C7orf25, GLI3, INHBA, INHBA-AS1, MRPL32, PSMA2 | | |
| chr7:45,197,383-55,756,780 | Gain | 10559,4 | p13 - p11.2 | ABCA13, ADCY1, C7orf57, C7orf65, C7orf69, C7orf72, CDC14C, COBL, DDC, EGFR, EGFR-AS1, FIGNL1, FKBP9L, FLJ45974, GRB10, HPVC1, HUS1, IGFBP1, IGFBP3, IKZF1, LANCL2, LINC00525, LOC100129427, LOC285878, PKD1L1, POM121L12, SEC61G, SEPT7P2, SUN3, TNS3, UPP1, VOPP1, VSTM2A, VWC2, ZPBP | | IKZF1, EGFR |
| chr7:68,579,533-68,942,882 | Gain | 363,35 | q11.22 | AUTS2 | | |
| chr7:71,677,963-75,945,347 | Gain | 4267,385 | q11.22 - q11.23 | ABHD11, ABHD11-AS1, BAZ1B, BCL7B, CCL24, CCL26, CLDN3, CLDN4, CLIP2, DNAJC30, DTX2, EIF4H, ELN, FDPSL2A, FKBP6, FZD9, GATSL1, GATSL2, GTF2I, GTF2IP1, GTF2IRD1, GTF2IRD2, GTF2IRD2B, GTF2IRD2P1, HIP1, HSPB1, LAT2, LIMK1, LOC100093631, LOC541473, MDH2, MIR4284, MIR4650-1, MIR4650-2, MIR4651, MIR590, MLXIPL, NCF1, NCF1B, NCF1C, NSUN5, NSUN5P1, NSUN5P2, PMS2L2, PMS2P3, PMS2P5, POM121, POM121C, POR, RFC2, RHBDD2, SBDSP1, SNORA14A, SPDYE5, SPDYE7P, SPDYE8P, SRCRB4D, SRRM3, STAG3L1, STAG3L2, STAG3L3, STX1A, STYXL1, TBL2, TMEM120A, TRIM50, TRIM73, TRIM74, TYW1B, VPS37D, WBSCR16, WBSCR22, WBSCR27, WBSCR28, YWHAG, ZP3 | hsa-mir-590 | ELN, HIP1 |

| Chromosome Region (hg18) | Event | Length (Kb) | Cytoband | Genes | miRNAs | Cancer Genes (Cancer Gene Census) |
|---|---|---|---|---|---|---|
| chr7:101,904,918-102,096,550 | Gain | 191,633 | q22.1 | POLR2J, POLR2J2, POLR2J3, RASA4, SPDYE2, SPDYE2L, UPK3BL | | |
| chr8:130,770,082-130,773,707 | Loss | 3,626 | q24.21 | | | |
| chr9:1,022,409-5,999,912 | Loss | 4977,504 | p24.3 - p24.1 | AK3, CD274, CDC37L1, DMRT2, ERMP1, FLJ35024, GLIS3, GLIS3-AS1, INSL4, INSL6, JAK2, KCNV2, KIAA0020, KIAA1432, KIAA2026, MIR101-2, MIR4665, MLANA, PDCD1LG2, PLGRKT, PPAPDC2, RCL1, RFX3, RLN1, RLN2, SLC1A1, SMARCA2, SPATA6L, VLDLR | hsa-mir-101-2 | JAK2, CD274 |
| chr9:6,067,101-6,701,708 | Gain | 634,608 | p24.1 | GLDC, IL33, TPD52L3, UHRF2 | | |
| chr9:7,034,677-8,006,161 | Loss | 971,485 | p24.1 | C9orf123, KDM4C | | |
| chr9:11,108,658-12,979,790 | Loss | 1871,133 | p23 | LURAP1L, TYRP1 | | |
| chr9:33,840,532-34,223,117 | Gain | 382,586 | p13.3 | DCAF12, SNORD121A, SNORD121B, UBAP1, UBAP2, UBE2R2 | | |
| chr9:34,803,277-35,688,747 | Gain | 885,471 | p13.3 | ARHGEF39, ATP8B5P, C9orf131, CA9, CCDC107, CD72, DNAJB5, FAM166B, FAM205B, FAM214B, FANCG, KIAA1045, MIR4667, PIGO, RMRP, RUSC2, SIT1, STOML2, TESK1, TLN1, TPM2, UNC13B, VCP | | FANCG |
| chr9:69,472,934-69,716,786 | Loss | 243,853 | q12 | CBWD3, CBWD5, FOXD4L2, FOXD4L4 | | |
| chr9:119,007,989-119,543,442 | Loss | 535,454 | q33.1 | ASTN2, TLR4 | | |
| chr9:126,700,419-127,058,353 | Gain | 357,935 | q33.3 | GOLGA1, HSPA5, PPP6C, RABEPK, SCAI | | |
| chr9:129,237,457-133,568,096 | Gain | 4330,64 | q33.3 - q34.13 | ABL1, AIF1L, AK1, ASB6, ASS1, C9orf106, C9orf114, C9orf117, C9orf16, C9orf50, C9orf78, CCBL1, CDK9, CERCAM, CIZ1, COQ4, CRAT, DNM1, DOLK, DOLPP1, DPM2, ENDOG, ENG, EXOSC2, FAM102A, FAM129B, FAM73B, FAM78A, FIBCD1, FNBP1, FPGS, FUBP3, GLE1, GOLGA2, GPR107, IER5L, LAMC3, LCN2, LOC100272217, LOC100289019, LOC100506100, LOC100506190, LOC389791, LRRC8A, LRSAM1, MIR199B, MIR219-2, MIR2861, MIR2964A, MIR3154, MIR3911, MIR3960, MIR4672, NAIF1, NCS1, NTMT1, NUP188, NUP214, ODF2, PHYHD1, PIP5KL1, PKN3, POMT1, PPAPDC3, PPP2R4, PRDM12, PRRC2B, PRRX2, PTGES, PTGES2, PTRH1, QRFP, RAPGEF1, RPL12, SET, SH2D3C, SH3GLB2, SLC25A25, SLC27A4, SNORA65, SNORD62A, SNORD62B, SPTAN1, ST6GALNAC4, ST6GALNAC6, STXBP1, SWI5, TBC1D13, TOR1A, TOR1B, TOR2A, TRUB2, TTC16, UCK1, URM1, USP20, WDR34, ZDHHC12, ZER1, ZNF79 | hsa-mir-199b, hsa-mir-219-2 | SET, ABL1, NUP214 |
| chr10:11,681,203-12,670,334 | Gain | 989,132 | p14 - p13 | C10orf47, CAMK1D, CDC123, DHTKD1, ECHDC3, LOC219731, MIR4480, NUDT5, SEC61A2, UPF2, USP6NL | | |
| chr10:60,517,126-64,452,930 | Loss | 3935,805 | q21.1 - q21.2 | ADO, ANK3, ARID5B, C10orf107, C10orf40, CCDC6, CDK1, EGR2, FAM13C, M1, MIR548AV, PHYHIPL, RHOBTB1, RTKN2, SLC16A9, TMEM26, ZNF365 | | CCDC6 |

| Chromosome Region (hg18) | Event | Length (Kb) | Cytoband | Genes | miRNAs | Cancer Genes (Cancer Gene Census) |
|---|---|---|---|---|---|---|
| chr10:114,556,933-120,300,754 | Loss | 5743,822 | q25.2 - q26.11 | ABLIM1, ADRB1, AFAP1L2, ATRNL1, C10orf118, C10orf82, CASC2, CASP7, CCDC172, DCLRE1A, EMX2, EMX2OS, ENO4, FAM160B1, FAM204A, GFRA1, HABP2, HSPA12A, KCNK18, KIAA1598, MIR2110, MIR3663, MIR4483, NHLRC2, NRAP, PDZD8, PLEKHS1, PNLIP, PNLIPRP1, PNLIPRP2, PNLIPRP3, RAB11FIP2, SLC18A2, TCF7L2, TDRD1, TRUB1, VAX1, VTI1A, VWA2 | hsa-mir-2110 | |
| chr10:121,972,874-135,235,140 | Loss | 13262,27 | q26.12 - q26.3 | ACADSB, ADAM12, ADAM8, ARMS2, ATE1, BCCIP, BNIP3, BTBD16, BUB3, C10orf120, C10orf137, C10orf88, C10orf90, C10orf91, CALY, CHST15, CLRN3, CPXM2, CTAGE7P, CTBP2, CUZD1, CYP2E1, DHX32, DMBT1, DOCK1, DPYSL4, EBF3, ECHS1, FAM175B, FAM196A, FAM24A, FAM24B, FAM24B-CUZD1, FAM53B, FANK1, FGFR2, FLJ37035, FLJ46300, FLJ46361, FOXI2, FUOM, GLRX3, GPR123, GPR26, HMX2, HMX3, HTRA1, IKZF5, INPP5A, JAKMIP3, KNDC1, LHPP, LOC100169752, LOC283038, LOC387723, LOC399815, LOC399829, LOC619207, LRRC27, METTL10, MGMT, MIR202, MIR378C, MIR3941, MIR3944, MIR4296, MIR4297, MIR4484, MIR5694, MKI67, MMP21, MTG1, NKX1-2, NKX6-2, NPS, NSMCE4A, OAT, PAOX, PLEKHA1, PPAPDC1A, PPP2R2D, PRAP1, PSTK, PTPRE, PWWP2B, SPRN, SPRNP1, STK32C, SYCE1, TACC2, TCERG1L, TEX36, TTC40, TUBGCP2, UROS, UTF1, VENTX, WDR11, WDR11-AS1, ZNF511, ZRANB1 | hsa-mir-202 | FGFR2 |
| chr11:118,472,904-118,662,946 | Gain | 190,043 | q23.3 | ABCG4, C2CD2L, CBL, CCDC153, DPAGT1, HINFP, NLRX1, PDZD3 | | CBL |
| chr11:133,635,039-133,733,087 | Gain | 98,049 | q25 | ACAD8, GLB1L2, GLB1L3 | | |
| chr12:130,574,940-132,283,344 | Gain | 1708,405 | q24.33 | ANKLE2, CHFR, DDX51, EP400, EP400NL, FBRSL1, GALNT9, GOLGA3, LOC100130238, LRCOL1, MMP17, NOC4L, P2RX2, PGAM5, POLE, PUS1, PXMP2, SFSWAP, SNORA49, ULK1, ZNF10, ZNF140, ZNF26, ZNF268, ZNF605, ZNF84 | | |
| chr13:49,696,818-50,350,033 | Loss | 653,216 | q14.3 | DLEU7, DLEU7-AS1 | | |
| chr13:63,276,254-63,629,038 | Loss | 352,785 | q21.31 | | | |
| chr13:97,442,160-97,535,532 | Gain | 93,373 | q32.2 | IPO5 | | |
| chr14:19,359,996-19,552,750 | Loss | 192,755 | q11.2 | OR4K1, OR4K14, OR4K15, OR4K2, OR4K5, OR4N2 | | |
| chr14:70,901,834-71,005,628 | Gain | 103,795 | q24.2 | SNORD56B | | |
| chr14:72,516,904-73,822,148 | Gain | 1305,245 | q24.2 - q24.3 | ABCD4, ACOT1, ACOT2, ACOT4, ACOT6, ALDH6A1, C14orf169, CCDC176, COQ6, DNAL1, ELMSAN1, ENTPD5, FAM161B, HEATR4, LIN52, MIR4505, NUMB, PAPLN, PNMA1, PSEN1, PTGR2, RBM25, VSX2, ZFYVE1, ZNF410 | | |
| chr14:76,238,797-77,461,704 | Gain | 1222,908 | q24.3 | ADCK1, AHSA1, ALKBH1, ANGEL1, C14orf166B, C14orf178, GSTZ1, IRF2BPL, ISM2, KIAA1737, MIR1260A, NGB, NOXRED1, POMT2, SAMD15, SLIRP, SNW1, SPTLC2, TMED8, TMEM63C, VASH1, VIPAS39, ZDHHC22 | hsa-mir-1260 | |

| Chromosome Region (hg18) | Event | Length (Kb) | Cytoband | Genes | miRNAs | Cancer Genes (Cancer Gene Census) |
|---|---|---|---|---|---|---|
| chr14:101,445,526-103,447,259 | Gain | 2001,734 | q32.31 - q32.33 | AMN, ANKRD9, APOPT1, BAG5, CDC42BPB, CINP, CKB, DYNC1H1, EIF5, EXOC3L4, HSP90AA1, KLC1, LINC00605, LINC00637, MARK3, MIR4309, MOK, PPP1R13B, PPP2R5C, RCOR1, SNORA28, TECPR2, TNFAIP2, TRAF3, TRMT61A, WDR20, XRCC3, ZFYVE21, ZNF839 | | HSP90AA1 |
| chr14:104,351,335-105,357,226 | Gain | 1005,892 | q32.33 | AHNAK2, BRF1, BTBD6, C14orf79, C14orf80, CDCA4, CRIP1, CRIP2, ELK2AP, GPR132, JAG2, KIAA0284, LINC00638, MTA1, NUDT14, PACS2, PLD4, TEX22, TMEM121 | | |
| chr15:92,632,460-99,520,942 | Loss | 6888,483 | q26.2 - q26.3 | ADAMTS17, ALDH1A3, ARRDC4, ASB7, CERS3, DNM1P46, FAM169B, FLJ42289, HSP90B2P, IGF1R, LINS, LOC145820, LOC400456, LOC91948, LRRC28, LRRK1, LYSMD4, MCTP2, MEF2A, MIR1469, MIR4714, NR2F2, PGPEP1L, PRKXP1, SPATA8, SYNM, TTC23 | hsa-mir-1469 | |
| chr16:11,247,099-11,979,901 | Gain | 732,803 | p13.13 | BCAR4, GSPT1, LITAF, PRM1, PRM2, PRM3, RMI2, RSL1D1, SNN, SNX29, SOCS1, TNFRSF17, TNP2, TXNDC11, ZC3H7A | hsa-mir-548h-2 | SOCS1, RMI2, TNFRSF17, RUNDC2A |
| chr16:87,945,758-87,986,817 | Gain | 41,06 | q24.3 | ANKRD11 | | |
| chr17:19,003,417-19,075,717 | Gain | 72,301 | p11.2 | | | |
| chr17:28,174,436-29,809,172 | Loss | 1634,737 | q11.2 - q12 | AA06, ASIC2, CCL1, CCL11, CCL13, CCL2, CCL7, CCL8, MIR548AC, MYO1D, SPACA3, TMEM98 | | |
| chr17:31,455,585-31,887,361 | Gain | 431,777 | q12 | CCL3L1, CCL3L3, CCL4, CCL4L1, CCL4L2, TBC1D3B, TBC1D3C, TBC1D3F, TBC1D3G, TBC1D3H | | |
| chr17:33,334,831-33,658,061 | Gain | 323,231 | q12 | LOC440434, TBC1D3, TBC1D3F | | |
| chr17:41,541,432-41,720,952 | Gain | 179,521 | q21.31 | KANSL1, KANSL1-AS1 | | |
| chr17:47,059,820-48,929,241 | Loss | 1869,422 | q21.33 - q22 | C17orf112, CA10 | | |
| chr18:6,177,548-7,953,566 | Gain | 1776,019 | p11.31 - p11.23 | ARHGAP28, L3MBTL4, LAMA1, LINC00668, LOC100130480, LRRC30, MIR4317, PTPRM | | |
| chr19:8,805,762-11,345,918 | Gain | 2540,157 | p13.2 | ANGPTL6, AP1M2, ATG4D, C19orf38, C19orf52, C19orf66, C19orf80, C3P1, CARM1, CCDC159, CDC37, CDKN2D, COL5A3, DNM2, DNMT1, DOCK6, EIF3G, FBXL12, FDX1L, ICAM1, ICAM3, ICAM4, ICAM5, ILF3, ILF3-AS1, KANK2, KEAP1, KRI1, LDLR, LOC284385, LPPR2, MBD3L1, MIR1181, MIR1238, MIR199A1, MIR4322, MIR4748, MIR638, MRPL4, MUC16, OLFM2, OR1M1, OR7D2, OR7D4, OR7E24, OR7G1, OR7G2, OR7G3, P2RY11, PDE4A, PIN1, PPAN, PPAN-P2RY11, QTRT1, RAB3D, RAVER1, RDH8, S1PR2, S1PR5, SLC44A2, SMARCA4, SNORD105, SNORD105B, SPC24, TMED1, TMEM205, TSPAN16, TYK2, UBL5, YIPF2, ZGLP1, ZNF121, ZNF177, ZNF266, ZNF317, ZNF426, ZNF559, ZNF559-ZNF177, ZNF560, ZNF561, ZNF562, ZNF699, ZNF812, ZNF846 | hsa-mir-638, hsa-mir-199a-1, hsa-mir-1238, hsa-mir-1181 | SMARCA4 |
| chr19:41,216,001-41,528,195 | Gain | 312,195 | q13.12 | CAPNS1, COX7A1, LINC00665, LOC100134317, OVOL3, POLR2I, TBCB, THAP8, WDR62, ZFP14, ZNF146, ZNF565 | | |
| chr20:13,894,196-13,988,218 | Gain | 94,023 | p12.1 | MACROD2, SEL1L2 | | |

| Chromosome Region (hg18) | Event | Length (Kb) | Cytoband | Genes | miRNAs | Cancer Genes (Cancer Gene Census) |
|---|---|---|---|---|---|---|
| chr20:13,925,326-14,968,466 | Loss | 1043,141 | p12.1 | FLRT3, MACROD2, MACROD2-AS1 | | |
| chr20:22,319,521-22,563,013 | Loss | 243,493 | p11.21 | FOXA2, LINC00261, LOC284788 | | |
| chr20:33,361,057-35,185,135 | Gain | 1824,079 | q11.22 - q11.23 | AAR2, C20orf118, C20orf132, C20orf173, C20orf24, CEP250, CNMPD1, CPNE1, DLGAP4, DSN1, EPB41L1, ERGIC3, FER1L4, GDF5, LINC00657, MYL9, NDRG3, NFS1, PHF20, RBL1, RBM12, RBM39, ROMO1, SAMHD1, SCAND1, SLA2, SOGA1, SPAG4, TGIF2, TGIF2-C20ORF24, UQCC | hsa-mir-1289-1 | |
| chr20:45,312,208-45,556,019 | Gain | 243,812 | q13.12 | LOC100131496, ZMYND8 | | |
| chr20:47,014,674-47,315,646 | Gain | 300,973 | q13.13 | ARFGEF2, CSE1L, DDX27, STAU1, ZNFX1 | | |
| chr20:48,701,717-50,206,769 | Gain | 1505,053 | q13.13 - q13.2 | ADNP, ATP9A, BCAS4, DPM1, KCNG1, MIR3194, MOCS3, NFATC2, PARD6B, SALL4, ZFP64 | | |
| chr20:51,475,357-51,856,995 | Gain | 381,639 | q13.2 | TSHZ2, ZNF217 | | |
| chr20:54,351,788-54,931,221 | Gain | 579,434 | q13.2 - q13.31 | AURKA, CASS4, CSTF1, FAM209A, FAM209B, FAM210B, GCNT7, RTFDC1, TFAP2C | | |
| chr21:14,424,909-26,218,975 | Loss | 11794,07 | q11.2 - q21.3 | ABCC13, APP, ATP5J, BTG3, C21orf37, C21orf91, C21orf91-OT1, CHODL, CHODL-AS1, CXADR, D21S2088E, GABPA, HSPA13, JAM2, LINC00158, LINC00308, LINC00317, LINC00320, LINC00478, LINC00515, LIPI, LOC339622, LOC388813, MIR125B2, MIR155, MIR155HG, MIR99A, MIRLET7C, MRPL39, NCAM2, NRIP1, RBM11, RNU6-67, SAMSN1, SAMSN1-AS1, TMPRSS15, USP25 | hsa-mir-125b-2, hsa-mir-99a, hsa-let-7c, hsa-mir-155 | |
| chr21:26,799,634-29,120,748 | Loss | 2321,115 | q21.3 | ADAMTS1, ADAMTS5, CYYR1, LINC00113, LINC00161, LINC00314, MIR4759, MIR5009 | | |
| chr21:39,802,029-46,907,149 | Loss | 7105,121 | q22.2 - q22.3 | ABCG1, ADARB1, AGPAT3, AIRE, B3GALT5, BACE2, C21orf128, C21orf2, C21orf33, C21orf58, C21orf67, C21orf88, C21orf90, C2CD2, CBS, COL18A1, COL18A1-AS1, COL18A1-AS2, COL6A1, COL6A2, CRYAA, CSTB, DIP2A, DIP2A-IT1, DNMT3L, DSCAM, DSCAM-AS1, DSCAM-IT1, FAM207A, FAM3B, FTCD, HSF2BP, ICOSLG, IGSF5, ITGB2, ITGB2-AS1, KRTAP10-1, KRTAP10-10, KRTAP10-11, KRTAP10-12, KRTAP10-2, KRTAP10-3, KRTAP10-4, KRTAP10-5, KRTAP10-6, KRTAP10-7, KRTAP10-8, KRTAP10-9, KRTAP12-1, KRTAP12-2, KRTAP12-3, KRTAP12-4, LINC00111, LINC00112, LINC00162, LINC00163, LINC00313, LINC00319, LINC00323, LINC00479, LOC100129027, LOC284837, LOC642852, LRRC3, LRRC3-AS1, LSS, MCM3AP, MCM3AP-AS1, MIR3197, MIR4760, MX1, MX2, NDUFV3, PCBP3, PCNT, PCP4, PDE9A, PDXK, PFKL, PKNOX1, PLAC4, POFUT2, PRDM15, PRMT2, PTTG1IP, PWP2, RIPK4, RRP1, RRP1B, RSPH1, S100B, SH3BGR, SIK1, SLC19A1, SLC37A1, SPATC1L, SSR4P1, SUMO3, TFF1, TFF2, TFF3, TMPRSS2, TMPRSS3, TRAPPC10, TRPM2, TSPEAR, U2AF1, UBASH3A, UBE2G2, UMODL1, WDR4, YBEY, ZNF295, ZNF295-AS1 | | TMPRSS2 |
| chr22:14,763,133-15,918,297 | Loss | 1155,165 | q11.1 | ANKRD62P1-PARP4P3, CCT8L2, CECR7, GAB4, HSFY1P1, OR11H1, TPTEP1, XKR3 | | |

| Chromosome Region (hg18) | Event | Length (Kb) | Cytoband | Genes | miRNAs | Cancer Genes (Cancer Gene Census) |
|---|---|---|---|---|---|---|
| chr22:46,535,490-48,140,017 | Loss | 1604,528 | q13.31 - q13.32 | FAM19A5, LOC100128946, LOC284933, MIR3201, MIR4535 | | |
| chrX:6,329-229,611 | Loss | 223,283 | p22.33 | GTPBP6, PLCXD1, PPP2R3B | | |
| chrX:9,978,135-10,038,900 | Loss | 60,766 | p22.2 | WWC3 | | |
| chrX:10,272,377-10,370,692 | Loss | 98,316 | p22.2 | | | |
| chrX:36,644,120-36,972,467 | Loss | 328,348 | p21.1 | FAM47C, FTH1P18 | | |
| chrX:46,334,243-49,260,494 | Gain | 2926,252 | p11.3 - p11.23 | ARAF, CACNA1F, CCDC120, CCDC22, CDK16, CFP, CHST7, CXXC1P1, CXorf31, EBP, ELK1, ERAS, FOXP3, FTSJ1, GAGE1, GAGE10, GAGE12B, GAGE12C, GAGE12D, GAGE12E, GAGE12F, GAGE12G, GAGE12H, GAGE12I, GAGE12J, GAGE13, GAGE2A, GAGE2B, GAGE2C, GAGE2D, GAGE2E, GAGE4, GAGE5, GAGE6, GAGE7, GAGE8, GATA1, GLOD5, GPKOW, GRIPAP1, HDAC6, INE1, KCND1, LOC100133957, MAGIX, MIR4769, NDUFB11, OTUD5, PCSK1N, PHF16, PIM2, PLP2, PORCN, PPP1R3F, PQBP1, PRAF2, PRICKLE3, RBM10, RBM3, RGN, RP2, SLC35A2, SLC38A5, SLC9A7, SNORA11C, SPACA5, SPACA5B, SSX1, SSX3, SSX4, SSX4B, SSX5, SSX6, SSX9, SUV39H1, SYN1, SYP, TBC1D25, TFE3, TIMM17B, TIMP1, UBA1, USP11, UXT, WAS, WDR13, WDR45, ZNF157, ZNF182, ZNF41, ZNF630, ZNF81 | | SSX1, SSX4, WAS, GATA1, TFE3 |
| chrX:52,954,827-54,604,813 | Gain | 1649,987 | p11.22 | FAM120C, FAM156A, FAM156B, FGD1, GNL3L, GPR173, HSD17B10, HUWE1, IQSEC2, KDM5C, MIR5684, MIR98, MIRLET7F2, PHF8, RIBC1, SMC1A, TSPYL2, TSR2, WNK3 | hsa-mir-98, hsa-let-7f-2 | KDM5C |
| chrX:55,504,304-55,508,361 | Loss | 4,058 | p11.21 | | | |
| chrX:72,137,133-72,217,950 | Gain | 80,818 | q13.1 - q13.2 | PABPC1L2A, PABPC1L2B | | |
| chrX:154,599,488-154,894,377 | Loss | 294,89 | q28 | IL9R, SPRY3, VAMP7 | | |
| chrY:6,181,134-6,401,059 | Loss | 219,926 | p11.2 | TTTY1, TTTY1B, TTTY2, TTTY21, TTTY21B, TTTY2B, TTTY7, TTTY7B, TTTY8, TTTY8B | | |
| chrY:7,488,962-10,358,451 | Loss | 2869,49 | p11.2 | FAM197Y2, FAM197Y5, RBMY1A3P, RBMY3AP, TSPY1, TSPY3, TSPY4, TSPY8, TTTY1, TTTY11, TTTY12, TTTY16, TTTY18, TTTY19, TTTY1B, TTTY2, TTTY20, TTTY21, TTTY21B, TTTY22, TTTY23, TTTY23B, TTTY2B, TTTY7, TTTY7B, TTTY8, TTTY8B | | |
| chrY:13,217,989-16,313,509 | Loss | 3095,521 | q11.21 - q11.221 | DDX3Y, NLGN4Y, NLGN4Y-AS1, TMSB4Y, TTTY15, USP9Y, UTY, VCY, VCY1B | | |
| chrY:16,504,762-18,416,664 | Loss | 1911,903 | q11.221 | FAM224A, FAM224B, FAM41AY1, FAM41AY2, XKRY, XKRY2 | | |
| chrY:18,418,194-18,701,486 | Loss | 283,293 | q11.221 | CDY2A, CDY2B | | |
| chrY:18,731,892-23,684,657 | Loss | 4952,766 | q11.221 - q11.223 | BCORP1, BPY2, BPY2B, BPY2C, CD24, EIF1AY, FAM224A, FAM224B, FAM41AY1, FAM41AY2, HSFY1, HSFY2, KDM5D, NCRNA00185, PRY, PRY2, RBMY1A1, RBMY1B, RBMY1D, RBMY1E, RBMY1F, RBMY1J, RBMY2EP, RBMY2FP, RPS4Y2, TTTY10, TTTY13, TTTY14, TTTY17A, TTTY17B, TTTY17C, TTTY5, TTTY6, TTTY6B, TTTY9A, TTTY9B, TXLNG2P, XKRY, XKRY2 | | |

| Chromosome Region (hg18) | Event | Length (Kb) | Cytoband | Genes | miRNAs | Cancer Genes (Cancer Gene Census) |
|---|---|---|---|---|---|---|
| chrY:23,885,207-25,323,292 | Loss | 1438,086 | q11.223 | BPY2, BPY2B, BPY2C, CDY1, CDY1B, CSPG4P1Y, DAZ3, DAZ4, GOLGA2P2Y, GOLGA2P3Y, TTTY17A, TTTY17B, TTTY17C, TTTY3, TTTY3B, TTTY4, TTTY4B, TTTY4C | | |
| chrY:25,504,095-26,742,672 | Loss | 1238,578 | q11.23 | BPY2, BPY2B, BPY2C, CDY1, CDY1B, CSPG4P1Y, GOLGA2P2Y, GOLGA2P3Y, TTTY17A, TTTY17B, TTTY17C, TTTY3, TTTY3B | | |

**Table S4.** Array-CGH analysis comparing ponatinib-resistant UT-7-315 clones A23 (with MS-5) *vs* C9 (without MS-5).

| Chromosome Region (hg18) | Event | Length (Kb) | Cytoband | Genes | miRNAs | Cancer Genes (Cancer Gene Census) |
|---|---|---|---|---|---|---|
| chr1:115,154,338-115,172,381 | Loss | 18,044 | p13.2 | | | |
| chr1:115,452,228-115,479,732 | Loss | 27,505 | p13.2 | | | |
| chr1:115,503,351-116,919,312 | Gain | 1415,962 | p13.2 - p13.1 | ATP1A1, ATP1A1OS, CASQ2, CD58, IGSF3, MAB21L3, NGF, NHLH2, SLC22A15, VANGL1 | | |
| chr1:159,749,160-159,891,063 | Gain | 141,904 | q23.3 | FCGR2A, FCGR2C, FCGR3A, FCGR3B, HSPA6, HSPA7 | | FCGR2B |
| chr1:197,147,959-198,181,788 | Gain | 1033,83 | q31.3 - q32.1 | LOC100131234 | | |
| chr1:204,404,417-205,354,108 | Loss | 949,692 | q32.1 - q32.2 | C1orf116, C1orf186, C4BPA, C4BPB, CTSE, DYRK3, EIF2D, FAIM3, FCAMR, IKBKE, IL10, IL19, IL20, IL24, MAPKAPK2, PFKFB2, PIGR, RASSF5, SRGAP2, YOD1 | | |
| chr1:229,220,576-229,774,281 | Loss | 553,706 | q42.2 | C1orf131, EGLN1, EXOC8, FAM89A, GNPAT, LOC149373, MIR1182, SNRPD2P2, SPRTN, TRIM67, TSNAX, TSNAX-DISC1 | hsa-mir-1182 | |
| chr1:245,617,250-247,171,256 | Loss | 1554,007 | q44 | GCSAML, GCSAML-AS1, LYPD8, MIR3124, NLRP3, OR11L1, OR13G1, OR14A16, OR14C36, OR14I1, OR1C1, OR2AK2, OR2B11, OR2C3, OR2G2, OR2G3, OR2G6, OR2L13, OR2L1P, OR2L2, OR2L3, OR2L5, OR2L8, OR2M1P, OR2M2, OR2M3, OR2M4, OR2M5, OR2M7, OR2T1, OR2T10, OR2T11, OR2T12, OR2T2, OR2T27, OR2T29, OR2T3, OR2T33, OR2T34, OR2T35, OR2T4, OR2T5, OR2T6, OR2T8, OR2W3, OR2W5, OR6F1, PGBD2, SH3BP5L, TRIM58, ZNF672, ZNF692 | | |
| chr2:45,857-1,257,265 | Gain | 1211,409 | p25.3 | ACP1, FAM150B, LOC339822, SH3YL1, SNTG2, TMEM18 | | |
| chr2:1,500,891-1,586,297 | Gain | 85,407 | p25.3 | TPO | | |
| chr2:11,507,501-11,558,540 | Loss | 51,04 | p25.1 | E2F6 | | |
| chr2:169,500,037-169,681,406 | Gain | 181,37 | q31.1 | ABCB11, DHRS9 | | |
| chr3:173,711,933-174,194,891 | Loss | 482,959 | q26.31 | ECT2, NCEH1, SPATA16, TNFSF10 | | |
| chr4:148,887,669-151,395,065 | Loss | 2507,397 | q31.23 - q31.3 | ARHGAP10, DCLK2, MIR4799, NR3C2 | | |
| chr5:132,596,984-132,617,409 | Gain | 20,426 | q31.1 | FSTL4 | | |

| Chromosome Region (hg18) | Event | Length (Kb) | Cytoband | Genes | miRNAs | Cancer Genes (Cancer Gene Census) |
|---|---|---|---|---|---|---|
| chr6:53,021,823-88,576,214 | Gain | 35554,39 | p12.1 - q15 | AKIRIN2, B3GAT2, BAG2, BAI3, BCKDHB, BEND6, BMP5, C6orf147, C6orf162, C6orf163, C6orf164, C6orf165, C6orf57, C6orf7, CD109, CGA, COL12A1, COL19A1, COL21A1, COL9A1, COX7A2, CYB5R4, DDX43, DOPEY1, DPPA5, DST, EEF1A1, ELOVL4, ELOVL5, EYS, FAM135A, FAM46A, FAM83B, FBXO9, FILIP1, GCLC, GCM1, GFRAL, GJB7, GUSBP4, HCRTR2, HMGCLL1, HMGN3, HTR1B, HTR1E, IBTK, ICK, IMPG1, IRAK1BP1, KCNQ5, KCNQ5-AS1, KHDC1, KHDC1L, KHDC3L, KHDRBS2, KIAA1009, KIAA1586, KLHL31, LCA5, LGSN, LINC00472, LMBRD1, LOC100288198, LOC100506804, LOC441155, LRRC1, MB21D1, ME1, MIR30A, MIR30C2, MIR4282, MIR548AD, MIR5685, MLIP, MLIP-IT1, MRAP2, MTO1, MYO6, NT5E, OGFRL1, OOEP, ORC3, PGM3, PHF3, PHIP, PRIM2, PRSS35, PTP4A1, RAB23, RARS2, RIMS1, RIPPLY2, RNU6-71, RPS16P5, RWDD2A, SENP6, SH3BGRL2, SLC17A5, SLC25A51P1, SLC35A1, SMAP1, SNAP91, SNHG5, SNORD50A, SNORD50B, SNX14, SYNCRIP, TBX18, TINAG, TMEM30A, TPBG, TTK, UBE3D, ZNF292, ZNF451 | hsa-mir-30c-2, hsa-mir-30a | |
| chr6:106,239,409-135,318,005 | Gain | 29078,59 | q21 - q23.3 | AIM1, AKAP7, AKD1, ALDH8A1, AMD1, ARG1, ARHGAP18, ARMC2, ASF1A, ATG5, BEND3, BET3L, BRD7P3, C6orf170, C6orf203, C6orf225, C6orf58, CCDC162P, CD164, CDC40, CDK19, CENPW, CEP57L1, CEP85L, CLVS2, COL10A1, CTAGE9, CTGF, DCBLD1, DDO, DSE, ECHDC1, ENPP1, ENPP3, EPB41L2, EYA4, FABP7, FAM162B, FAM184A, FAM26D, FAM26E, FAM26F, FIG4, FLJ34503, FOXO3, FRK, FYN, GJA1, GOPC, GPR6, GPRC6A, GSTM2P1, GTF3C6, HDAC2, HDDC2, HEY2, HINT3, HMGA1P7, HS3ST5, HSF2, KIAA0408, KIAA1919, KPNA5, L3MBTL3, LACE1, LAMA2, LAMA4, LINC00222, LINC00326, LOC100287632, LOC100422737, LOC100507203, LOC100507254, LOC154092, LOC285758, LOC285762, LOC643623, MAN1A1, MARCKS, MCM9, MED23, METTL24, MGC34034, MICAL1, MIR548B, MOXD1, NCOA7, NKAIN2, NR2E1, NT5DC1, NUS1, OR2A4, OSTM1, PDSS2, PKIB, PLN, PPIL6, PRDM1, PTPRK, QRSL1, REV3L, RFPL4B, RFX6, RNF146, RNF217, ROS1, RPF2, RPS12, RSPH4A, RSPO3, RTN4IP1, RWDD1, SAMD3, SCML4, SEC63, SERINC1, SESN1, SGK1, SLC16A10, SLC18B1, SLC22A16, SLC2A12, SLC35F1, SMPD2, SMPDL3A, SNORA33, SNORD100, SNORD101, SNX3, SOBP, SOGA3, STL, STX7, TAAR1, TAAR2, TAAR3, TAAR5, TAAR6, TAAR8, TAAR9, TBPL1, TCF21, THEMIS, TMEM200A, TMEM244, TPD52L1, TPI1P3, TRAF3IP2, TRAF3IP2-AS1, TRDN, TRMT11, TSPYL1, TSPYL4, TUBE1, VGLL2, VNN1, VNN2, VNN3, WASF1, WISP3, ZBTB24, ZUFSP | hsa-mir-548b, hsa-mir-587, hsa-mir-588 | PRDM1, FOXO3, ROS1, GOPC |
| chr6:159,350,382-160,160,110 | Gain | 809,729 | q25.3 | ACAT2, FNDC1, LOC100129518, MRPL18, PNLDC1, SNORA20, SNORA29, SOD2, TAGAP, TCP1, WTAP | | |

| Chromosome Region (hg18) | Event | Length (Kb) | Cytoband | Genes | miRNAs | Cancer Genes (Cancer Gene Census) |
|---|---|---|---|---|---|---|
| chr7:1,941,326-2,155,122 | Loss | 213,797 | p22.3 - p22.2 | MAD1L1 | | |
| chr7:55,259,660-55,534,909 | Loss | 275,25 | p11.2 | LANCL2, VOPP1 | | |
| chr8:12,975,060-13,414,607 | Gain | 439,548 | p22 | DLC1 | | |
| chr8:118,965,914-119,714,487 | Gain | 748,574 | q24.11 - q24.12 | EXT1, SAMD12, SAMD12-AS1 | | EXT1 |
| chr8:130,458,786-130,637,003 | Gain | 178,218 | q24.21 | | | |
| chr9:33,972-227,197 | Gain | 193,226 | p24.3 | C9orf66, CBWD1, DOCK8, FOXD4 | | |
| chr9:2,496,958-2,523,497 | Loss | 26,54 | p24.2 | | | |
| chr9:4,029,367-9,825,448 | Loss | 5796,082 | p24.2 - p23 | AK3, C9orf123, CD274, CDC37L1, ERMP1, GLDC, GLIS3, IL33, INSL4, INSL6, JAK2, KDM4C, KIAA1432, KIAA2026, MIR101-2, MIR4665, MLANA, PDCD1LG2, PLGRKT, PPAPDC2, PTPRD, RANBP6, RCL1, RLN1, RLN2, SLC1A1, SPATA6L, TPD52L3, UHRF2 | hsa-mir-101-2 | JAK2, CD274 |
| chr9:11,108,658-12,529,977 | Loss | 1421,32 | p23 | | | |
| chr9:20,245,178-32,102,682 | Gain | 11857,50 | p21.3 - p21.1 | C9orf53, C9orf72, CAAP1, CDKN2A, CDKN2B, CDKN2B-AS1, DMRTA1, ELAVL2, EQTN, FLJ35282, FOCAD, IFNA1, IFNA10, IFNA13, IFNA14, IFNA16, IFNA17, IFNA2, IFNA21, IFNA22P, IFNA4, IFNA5, IFNA6, IFNA7, IFNA8, IFNB1, IFNE, IFNK, IFNW1, IFT74, IZUMO3, KLHL9, LINC00032, LINGO2, LOC100506422, LOC401497, LRRC19, MIR31, MIR31HG, MIR4473, MIR4474, MIR491, MIR873, MIR876, MLLT3, MOB3B, MTAP, PLAA, PTPLAD2, TEK, TUSC1 | hsa-mir-876, hsa-mir-31, hsa-mir-491, hsa-mir-873 | MLLT3, CDKN2A |
| chr9:32,004,034-32,102,682 | Loss | 98,649 | p21.1 | | | |
| chr10:128,605,650-128,701,435 | Loss | 95,786 | q26.2 | DOCK1 | | |
| chr13:51,415,888-51,441,856 | Loss | 25,969 | q14.3 | ATP7B | | |
| chr13:63,276,254-63,629,038 | Loss | 352,785 | q21.31 | | | |
| chr13:90,579,146-90,727,954 | Loss | 148,809 | q31.3 | LINC00379 | | |
| chr13:90,822,300-91,270,798 | Loss | 448,499 | q31.3 | GPC5 | | |
| chr14:18,407,780-19,359,996 | Gain | 952,217 | q11.1 - q11.2 | LOC642426, OR11H12, OR11H2, OR4M1, OR4Q3, POTEG, POTEM | | |
| chr14:24,784,036-24,901,016 | Loss | 116,981 | q12 | | | |
| chr16:77,713,183-77,795,795 | Gain | 82,613 | q23.1 | WWOX | | |
| chr17:31,455,585-31,887,361 | Gain | 431,777 | q12 | CCL3L1, CCL3L3, CCL4, CCL4L1, CCL4L2, TBC1D3B, TBC1D3C, TBC1D3F, TBC1D3G, TBC1D3H | | |
| chr17:33,334,831-33,658,061 | Gain | 323,231 | q12 | LOC440434, TBC1D3, TBC1D3F | | |

| Chromosome Region (hg18) | Event | Length (Kb) | Cytoband | Genes | miRNAs | Cancer Genes (Cancer Gene Census) |
|---|---|---|---|---|---|---|
| chr18:70,626,420-72,301,711 | Gain | 1675,292 | q22.3 - q23 | C18orf62, LOC339298, TSHZ1, ZADH2, ZNF407, ZNF516 | | |
| chr18:73,477,939-74,857,608 | Gain | 1379,67 | q23 | SALL3 | | |
| chr19:56,795,726-56,845,576 | Loss | 49,851 | q13.33 | SIGLEC14, SIGLEC5 | | |
| chr19:56,962,999-57,320,049 | Loss | 357,051 | q13.33 | FPR2, FPR3, ZNF350, ZNF432, ZNF577, ZNF613, ZNF614, ZNF615, ZNF616, ZNF649, ZNF841 | | |
| chr19:59,231,080-59,251,120 | Loss | 20,041 | q13.42 | VSTM1 | | |
| chr20:3,836,585-4,075,142 | Gain | 238,558 | p13 | MIR103A2, MIR103B2, PANK2, RNF24 | hsa-mir-103-2, hsa-mir-103-2-as | |
| chr21:17,572,446-17,686,293 | Loss | 113,848 | q21.1 | | | |
| chr21:18,041,169-29,079,923 | Gain | 11038,75 | q21.1 - q21.3 | ADAMTS1, ADAMTS5, APP, ATP5J, C21orf91, C21orf91-OT1, CHODL, CHODL-AS1, CYYR1, D21S2088E, GABPA, JAM2, LINC00113, LINC00158, LINC00161, LINC00308, LINC00314, LINC00317, LINC00320, LINC00515, LOC339622, MIR155, MIR155HG, MIR4759, MIR5009, MRPL39, NCAM2, RNU6-67, TMPRSS15 | hsa-mir-155 | |
| chr21:34,674,531-46,907,149 | Loss | 12232,61 | q22.11 - q22.3 | ABCG1, ADARB1, AGPAT3, AIRE, B3GALT5, BACE2, BRWD1, BRWD1-AS1, BRWD1-IT2, C21orf128, C21orf2, C21orf33, C21orf58, C21orf67, C21orf88, C21orf90, C2CD2, CBR1, CBR3, CBR3-AS1, CBS, CHAF1B, CLDN14, CLIC6, COL18A1, COL18A1-AS1, COL18A1-AS2, COL6A1, COL6A2, CRYAA, CSTB, DIP2A, DIP2A-IT1, DNMT3L, DOPEY2, DSCAM, DSCAM-AS1, DSCAM-IT1, DSCR10, DSCR3, DSCR4, DSCR6, DSCR8, DSCR9, DYRK1A, ERG, ETS2, FAM165B, FAM207A, FAM3B, FTCD, HLCS, HMGN1, HSF2BP, ICOSLG, IGSF5, ITGB2, ITGB2-AS1, KCNE1, KCNJ15, KCNJ6, KRTAP10-1, KRTAP10-10, KRTAP10-11, KRTAP10-12, KRTAP10-2, KRTAP10-3, KRTAP10-4, KRTAP10-5, KRTAP10-6, KRTAP10-7, KRTAP10-8, KRTAP10-9, KRTAP12-1, KRTAP12-2, KRTAP12-3, KRTAP12-4, LCA5L, LINC00111, LINC00112, LINC00114, LINC00160, LINC00162, LINC00163, LINC00313, LINC00319, LINC00323, LINC00479, LOC100129027, LOC100133286, LOC100506385, LOC284837, LOC642852, LRRC3, LRRC3-AS1, LSS, MCM3AP, MCM3AP-AS1, MIR3197, MIR4760, MIR802, MORC3, MX1, MX2, NDUFV3, PCBP3, PCNT, PCP4, PDE9A, PDXK, PFKL, PIGP, PKNOX1, PLAC4, POFUT2, PRDM15, PRMT2, PSMG1, PTTG1IP, PWP2, RCAN1, RIPK4, RRP1, RRP1B, RSPH1, RUNX1, RUNX1-IT1, S100B, SETD4, SH3BGR, SIK1, SIM2, SLC19A1, SLC37A1, SPATC1L, SSR4P1, SUMO3, TFF1, TFF2, TFF3, TMPRSS2, TMPRSS3, TRAPPC10, TRPM2, TSPEAR, TTC3, U2AF1, UBASH3A, UBE2G2, UMODL1, WDR4, WRB, YBEY, ZNF295, ZNF295-AS1 | hsa-mir-802 | RUNX1, ERG, TMPRSS2 |

| Chromosome Region (hg18) | Event | Length (Kb) | Cytoband | Genes | miRNAs | Cancer Genes (Cancer Gene Census) |
|---|---|---|---|---|---|---|
| chr22:24,852,634-24,887,626 | Gain | 34,993 | q12.1 | MIR1302-1 | | |
| chrX:918,399-921,332 | Loss | 2,934 | p22.33 | | | |
| chrX:3,553,206-3,678,990 | Gain | 125,785 | p22.33 | PRKX | | |
| chrX:55,950,543-55,967,087 | Gain | 16,545 | p11.21 | | | |
| chrX:90,059,469-90,356,371 | Loss | 296,903 | q21.31 | | | |
| chrX:90,416,005-90,756,769 | Gain | 340,765 | q21.31 | PABPC5 | | |
| chrY:918,399-926,844 | Loss | 8,446 | p11.32 | | | |
| chrY:22,636,421-23,058,870 | Loss | 422,45 | q11.223 | PRY, PRY2, RBMY1F, RBMY1J, RBMY2FP, TTTY5, TTTY6, TTTY6B | | |
| chrY:23,171,649-23,327,051 | Loss | 155,403 | q11.223 | | | |
| chrY:26,659,439-26,689,998 | Loss | 30,56 | q11.23 | | | |

**Table S5.** Array-CGH analysis comparing ponatinib-resistant UT-7-315 clones A24 (with MS-5) *vs* B4 (without MS-5).

| Chromosome Region (hg18) | Event | Length (Kb) | Cytoband | Genes | miRNAs | Cancer Genes (Cancer Gene Census) |
|---|---|---|---|---|---|---|
| chr1:113,878,387-113,995,349 | Gain | 116,963 | p13.2 | MAGI3 | | |
| chr1:115,154,338-115,172,381 | Loss | 18,044 | p13.2 | | | |
| chr1:115,503,351-116,910,628 | Gain | 1407,278 | p13.2 - p13.1 | ATP1A1, ATP1A1OS, CASQ2, CD58, MAB21L3, NGF, NHLH2, SLC22A15, VANGL1 | | |
| chr1:117,007,943-119,033,535 | Gain | 2025,593 | p13.1 - p12 | CD101, CD2, FAM46C, GDAP2, IGSF3, MAN1A2, MIR320B1, MIR942, PTGFRN, SPAG17, TRIM45, TTF2, VTCN1, WDR3 | hsa-mir-320b-1, hsa-mir-942 | |
| chr1:173,328,582-173,585,591 | Gain | 257,01 | q25.1 | KIAA0040, TNN, TNR | | |
| chr1:182,930,923-196,999,802 | Gain | 14068,88 | q25.3 - q31.3 | ASPM, ATP6V1G3, B3GALT2, C1orf27, C1orf53, CDC73, CFH, CFHR1, CFHR2, CFHR3, CFHR4, CFHR5, CRB1, DENND1B, EDEM3, F13B, FAM129A, FAM5C, GLRX2, HMCN1, IVNS1ABP, KCNT2, LHX9, LOC100288079, LOC440704, MIR1278, MIR4735, NEK7, OCLM, PDC, PLA2G4A, PRG4, PTGS2, PTPRC, RGS1, RGS13, RGS18, RGS2, RGS21, RNF2, RNU6-72, SWT1, TPR, TRMT1L, TROVE2, UCHL5, ZBTB41 | hsa-mir-1278 | TPR, CDC73 |
| chr1:197,147,959-197,395,503 | Gain | 247,545 | q31.3 | LOC100131234 | | |
| chr1:205,365,393-206,178,926 | Gain | 813,534 | q32.2 | C4BPA, CD34, CD46, CD55, CR1, CR1L, CR2, LOC148696, MIR29B2, MIR29C | hsa-mir-29c, hsa-mir-29b-2 | |
| chr1:229,211,041-229,446,737 | Gain | 235,697 | q42.2 | C1orf131, FAM89A, GNPAT, LOC149373, MIR1182, TRIM67 | hsa-mir-1182 | |
| chr1:241,050,211-241,183,178 | Loss | 132,968 | q43 | | | |
| chr1:245,715,340-247,171,256 | Gain | 1455,917 | q44 | GCSAML, GCSAML-AS1, LYPD8, MIR3124, OR11L1, OR13G1, OR14A16, OR14C36, OR14I1, OR1C1, OR2AK2, OR2C3, OR2G2, OR2G3, OR2G6, OR2L13, OR2L1P, OR2L2, OR2L3, OR2L5, OR2L8, OR2M1P, OR2M2, OR2M3, OR2M4, OR2M5, OR2M7, OR2T1, OR2T10, OR2T11, OR2T12, OR2T2, OR2T27, OR2T29, OR2T3, OR2T33, OR2T34, OR2T35, OR2T4, OR2T5, OR2T6, OR2T8, OR2W3, OR2W5, OR6F1, PGBD2, SH3BP5L, TRIM58, ZNF672, ZNF692 | | |
| chr2:85,959,401-86,096,136 | Loss | 136,736 | p11.2 | ST3GAL5 | | |
| chr2:152,303,950-152,899,220 | Loss | 595,271 | q23.3 | ARL5A, CACNB4, STAM2 | | |
| chr2:169,500,037-169,501,119 | Gain | 1,083 | q31.1 | ABCB11 | | |
| chr2:212,035,464-212,224,004 | Gain | 188,541 | q34 | ERBB4 | | |
| chr3:3,746,746-4,299,772 | Gain | 553,027 | p26.2 | LRRN1 | | |

| Chromosome Region (hg18) | Event | Length (Kb) | Cytoband | Genes | miRNAs | Cancer Genes (Cancer Gene Census) |
|---|---|---|---|---|---|---|
| chr3:87,927,361-88,199,556 | Loss | 272,196 | p11.2 | CGGBP1, HTR1F | | |
| chr3:173,711,933-174,194,891 | Loss | 482,959 | q26.31 | ECT2, NCEH1, SPATA16, TNFSF10 | | |
| chr4:132,751,038-133,195,125 | Gain | 444,088 | q28.3 | | | |
| chr5:134,320,277-137,784,356 | Loss | 3464,08 | q31.1 - q31.2 | BRD8, C5orf20, CATSPER3, CDC23, CDC25C, CXCL14, FAM13B, FAM53C, FBXL21, GFRA3, H2AFY, HNRNPA0, IL9, KDM3B, KIF20A, KLHL3, LECT2, LOC340073, LOC340074, LOC389332, MIR874, MYOT, NEUROG1, NME5, NPY6R, PCBD2, PITX1, PKD2L2, SLC25A48, SMAD5, SMAD5-AS1, SPOCK1, TGFBI, TIFAB, TRPC7, VTRNA2-1, WNT8A | hsa-mir-886, hsa-mir-874 | |
| chr6:2,625,415-2,752,897 | Loss | 127,483 | p25.2 | MYLK4, WRNIP1 | | |

| Chromosome Region (hg18) | Event | Length (Kb) | Cytoband | Genes | miRNAs | Cancer Genes (Cancer Gene Census) |
|---|---|---|---|---|---|---|
| chr6:41,252,762-101,898,646 | Loss | 60645,885 | p21.1 - q16.3 | AARS2, ABCC10, AKIRIN2, ANKRD6, ASCC3, ATP6V0CP3, B3GAT2, BACH2, BAG2, BAI3, BCKDHB, BEND6, BMP5, BYSL, C6orf108, C6orf132, C6orf141, C6orf147, C6orf162, C6orf163, C6orf164, C6orf165, C6orf223, C6orf226, C6orf57, C6orf7, CAPN11, CASP8AP2, CCNC, CCND3, CD109, CD2AP, CDC5L, CENPQ, CGA, CLIC5, CNPY3, CNR1, COL12A1, COL19A1, COL21A1, COL9A1, COQ3, COX7A2, CRIP3, CRISP1, CRISP2, CRISP3, CUL7, CUL9, CYB5R4, CYP39A1, DDX43, DEFB110, DEFB112, DEFB113, DEFB114, DEFB133, DLK2, DOPEY1, DPPA5, DST, EEF1A1, EFHC1, ELOVL4, ELOVL5, ENPP4, ENPP5, EPHA7, EYS, FAM135A, FAM46A, FAM83B, FAXC, FBXL4, FBXO9, FHL5, FILIP1, FOXP4, FRS3, FUT9, GABRR1, GABRR2, GCLC, GCM1, GFRAL, GJA10, GJB7, GLYATL3, GNMT, GPR110, GPR111, GPR115, GPR116, GPR63, GSTA1, GSTA2, GSTA3, GSTA4, GSTA5, GSTA7P, GTPBP2, GUCA1A, GUCA1B, GUSBP4, HCRTR2, HMGCLL1, HMGN3, HSP90AB1, HTR1B, HTR1E, IBTK, ICK, IL17A, IL17F, IMPG1, IRAK1BP1, KCNQ5, KCNQ5-AS1, KHDC1, KHDC1L, KHDC3L, KHDRBS2, KIAA0240, KIAA1009, KIAA1586, KLC4, KLHDC3, KLHL31, KLHL32, LCA5, LGSN, LINC00472, LMBRD1, LOC100132354, LOC100287718, LOC100288198, LOC100506804, LOC441155, LOC728012, LOC730101, LRRC1, LRRC73, LYRM2, MAD2L1BP, MANEA, MAP3K7, MB21D1, MCHR2, MCM3, MDFI, MDN1, ME1, MEA1, MED20, MEP1A, MIR133B, MIR206, MIR2113, MIR30A, MIR30C2, MIR4282, MIR4464, MIR4641, MIR4642, MIR4643, MIR4647, MIR548AD, MIR5685, MIR586, MLIP, MLIP-IT1, MMS22L, MRAP2, MRPL14, MRPL2, MRPS10, MRPS18A, MTO1, MUT, MYO6, NCR2, NDUFAF4, NFKBIE, NT5E, OGFRL1, OOEP, OPN5, ORC3, PAQR8, PEX6, PGC, PGK2, PGM3, PHF3, PHIP, PKHD1, PLA2G7, PM20D2, PNISR, PNRC1, POLH, POLR1C, POU3F2, PPP2R5D, PRDM13, PRICKLE4, PRIM2, PRPH2, PRSS35, PTCHD4, PTCRA, PTK7, PTP4A1, RAB23, RARS2, RCAN2, RHAG, RIMS1, RIPPLY2, RNGTT, RNU6-71, RPL7L1, RPS16P5, RRAGD, RRP36, RSPH9, RUNX2, RWDD2A, SENP6, SH3BGRL2, SIM1, SLC17A5, SLC22A7, SLC25A27, SLC25A51P1, SLC29A1, SLC35A1, SLC35B2, SMAP1, SNAP91, SNHG5, SNORD50A, SNORD50B, SNX14, SPACA1, SPATS1, SRF, SRSF12, SUPT3H, SYNCRIP, TAF8, TBCC, TBX18, TCTE1, TDRD6, TFAP2B, TFAP2D, TFEB, TINAG, TJAP1, TMEM14A, TMEM151B, TMEM30A, TMEM63B, TNFRSF21, TOMM6, TPBG, TRAM2, TREM1, TREML2, TREML3P, TREML4, TREML5P, TRERF1, TSG1, TSTD3, TTBK1, TTK, UBE2J1, UBE3D, UBR2, UFL1, USP45, USP49, VEGFA, XPO5, YIPF3, ZNF292, ZNF318, ZNF451 | hsa-mir-30c-2, hsa-mir-133b, hsa-mir-206, hsa-mir-586, hsa-mir-2113, hsa-mir-30a | TFEB, CCND3, HSP90AB1 |

| Chromosome Region (hg18) | Event | Length (Kb) | Cytoband | Genes | miRNAs | Cancer Genes (Cancer Gene Census) |
|---|---|---|---|---|---|---|
| chr7:130,978-2,155,122 | Gain | 2024,145 | p22.3 - p22.2 | ADAP1, C7orf50, COX19, CYP2W1, ELFN1, FAM20C, FLJ44511, GET4, GPER, GPR146, HEATR2, INTS1, MAD1L1, MAFK, MICALL2, MIR339, MIR4655, PDGFA, PRKAR1B, PSMG3, PSMG3-AS1, SUN1, TFAMP1, TMEM184A, UNCX, ZFAND2A | hsa-mir-339 | |
| chr8:1,750,526-1,814,248 | Gain | 63,723 | p23.3 | ARHGEF10, MIR596 | hsa-mir-596 | |
| chr8:2,055,105-2,209,932 | Gain | 154,828 | p23.3 - p23.2 | MYOM2 | | |
| chr8:118,965,914-119,714,487 | Gain | 748,574 | q24.11 - q24.12 | EXT1, SAMD12, SAMD12-AS1 | | EXT1 |
| chr8:130,358,880-130,773,707 | Gain | 414,828 | q24.21 | | | |
| chr9:2,496,958-2,523,497 | Gain | 26,54 | p24.2 | | | |
| chr9:5,380,368-5,999,912 | Loss | 619,545 | p24.1 | CD274, ERMP1, KIAA1432, KIAA2026, MIR4665, MLANA, PDCD1LG2, PLGRKT | | CD274 |
| chr9:8,096,536-8,167,385 | Gain | 70,85 | p24.1 | | | |
| chr9:8,374,089-9,825,448 | Gain | 1451,36 | p24.1 - p23 | PTPRD | | |
| chr9:12,529,977-12,979,790 | Gain | 449,814 | p23 | LURAP1L, TYRP1 | | |
| chr9:20,245,178-32,102,682 | Loss | 11857,505 | p21.3 - p21.1 | C9orf53, C9orf72, CAAP1, CDKN2A, CDKN2B, CDKN2B-AS1, DMRTA1, ELAVL2, EQTN, FLJ35282, FOCAD, IFNA1, IFNA10, IFNA13, IFNA14, IFNA16, IFNA17, IFNA2, IFNA21, IFNA22P, IFNA4, IFNA5, IFNA6, IFNA7, IFNA8, IFNB1, IFNE, IFNK, IFNW1, IFT74, IZUMO3, KLHL9, LINC00032, LINGO2, LOC100506422, LOC401497, LRRC19, MIR31, MIR31HG, MIR4473, MIR4474, MIR491, MIR873, MIR876, MLLT3, MOB3B, MTAP, PLAA, PTPLAD2, TEK, TUSC1 | hsa-mir-876, hsa-mir-31, hsa-mir-491, hsa-mir-873 | MLLT3, CDKN2A |
| chr9:102,097,962-102,884,174 | Loss | 786,213 | q31.1 | INVS, LPPR1, MSANTD3, MSANTD3-TMEFF1, MURC, TEX10, TMEFF1 | | |
| chr10:37,490,461-37,524,172 | Loss | 33,712 | p11.21 | ANKRD30A | | |
| chr10:60,517,126-64,979,704 | Loss | 4462,579 | q21.1 - q21.3 | ADO, ANK3, ARID5B, C10orf107, C10orf40, CCDC6, CDK1, EGR2, FAM13C, JMJD1C, LOC84989, M1, MIR1296, MIR548AV, NRBF2, PHYHIPL, REEP3, RHOBTB1, RTKN2, SLC16A9, TMEM26, ZNF365 | hsa-mir-1296 | CCDC6 |
| chr10:81,148,306-81,566,427 | Loss | 418,122 | q22.3 | LOC650623, SFTPA1, SFTPA2 | | |
| chr10:88,920,768-89,249,049 | Loss | 328,282 | q23.2 | FAM22A, FAM22D, FAM35A, LOC439994, LOC728190, LOC728218 | | |
| chr11:14,539,491-14,715,074 | Loss | 175,584 | p15.2 | PDE3B, PSMA1 | | |
| chr11:61,226,358-61,251,263 | Loss | 24,906 | q12.2 | DAGLA | | |
| chr12:103,480,720-103,527,717 | Gain | 46,998 | q23.3 | CHST11, MIR3922 | | |
| chr13:51,415,888-51,441,856 | Loss | 25,969 | q14.3 | ATP7B | | |
| chr14:18,407,780-19,359,996 | Loss | 952,217 | q11.1 - q11.2 | LOC642426, OR11H12, OR11H2, OR4M1, OR4Q3, POTEG, POTEM | | |

| Chromosome Region (hg18) | Event | Length (Kb) | Cytoband | Genes | miRNAs | Cancer Genes (Cancer Gene Census) |
|---|---|---|---|---|---|---|
| chr14:24,784,036-25,825,716 | Gain | 1041,681 | q12 | | | |
| chr16:14,899,883-15,030,993 | Loss | 131,111 | p13.11 | MIR1972-1, MIR1972-2, MIR3179-1, MIR3179-2, MIR3179-3, MIR3180-1, MIR3180-2, MIR3180-3, NPIP, PDXDC1 | hsa-mir-1972 | |
| chr16:68,559,335-68,749,192 | Loss | 189,858 | q22.1 | MIR1972-1, MIR1972-2, PDPR, PDXDC2P | hsa-mir-1972 | |
| chr16:77,492,443-77,510,238 | Gain | 17,796 | q23.1 | WWOX | | |
| chr17:41,541,432-41,720,952 | Loss | 179,521 | q21.31 | KANSL1, KANSL1-AS1 | | |
| chr17:42,037,190-42,121,317 | Loss | 84,128 | q21.32 | NSF, NSFP1 | | |
| chr19:56,950,190-59,313,839<br><br>Part1 | Loss | 2363,65 | q13.33 - q13.42 | BIRC8, CACNG6, CACNG7, CACNG8, DPRX, ERVV-1, ERVV-2, FAM90A27P, FPR2, FPR3, LOC284379, MIR1283-1, MIR1283-2, MIR1323, MIR371A, MIR371B, MIR372, MIR373, MIR498, MIR512-1, MIR512-2, MIR515-1, MIR515-2, MIR516A1, MIR516A2, MIR516B1, MIR516B2, MIR517A, MIR517B, MIR517C, MIR518A1, MIR518A2, MIR518B, MIR518C, MIR518D, MIR518E, MIR518F, MIR519A1, MIR519A2, MIR519B, MIR519C, MIR519D, MIR519E, MIR520A, MIR520B, MIR520C, MIR520D, MIR520E, MIR520F, MIR520G, MIR520H, MIR521-1, MIR521-2, MIR522, MIR523, MIR524, MIR525, MIR526A1, MIR526A2, MIR526B, MIR527, MIR643, MIR935, MYADM, NDUFA3, NLRP12, OSCAR, PPP2R1A, PRKCG, PRPF31, TARM1, TFPT, TPM3P9, VN1R2, VN1R4, VSTM1, ZNF137P, ZNF160, ZNF28, ZNF320, ZNF321P, ZNF331, ZNF347, ZNF350, ZNF415, ZNF432, ZNF468, ZNF480, ZNF525, ZNF528, ZNF534, ZNF577, ZNF578, ZNF600, ZNF610, ZNF611, ZNF613, ZNF614, ZNF615, ZNF616, ZNF649, ZNF665, ZNF677, ZNF701, ZNF702P, ZNF761, ZNF765, ZNF766, ZNF808, ZNF813, ZNF816, ZNF816-ZNF321P, ZNF818P, ZNF83, ZNF836, ZNF841, ZNF845, ZNF880 | hsa-mir-518f, hsa-mir-512-1, hsa-mir-519a-1, hsa-mir-512-2, hsa-mir-519a-2, hsa-mir-518a-1, hsa-mir-518a-2, hsa-mir-518b, hsa-mir-518c, hsa-mir-518d, hsa-mir-518e, hsa-mir-520g, hsa-mir-520h, hsa-mir-520e, hsa-mir-520f, hsa-mir-520b, hsa-mir-520a, hsa-mir-520d, hsa-mir-520c, hsa-mir-519b, hsa-mir-373, hsa-mir-515-1, hsa-mir-372, | |

| Chromosome Region (hg18) | Event | Length (Kb) | Cytoband | Genes | miRNAs | Cancer Genes (Cancer Gene Census) |
|---|---|---|---|---|---|---|
| chr19:56,950,190-59,313,839<br><br>Part2 | Loss | 2363,65 | q13.33 - q13.42 | | hsa-mir-519e, hsa-mir-519c, hsa-mir-519d, hsa-mir-522, hsa-mir-515-2, hsa-mir-1283-1, hsa-mir-525, hsa-mir-1283-2, hsa-mir-524, hsa-mir-523, hsa-mir-526a-1, hsa-mir-526a-2, hsa-mir-527, hsa-mir-643, hsa-mir-1323, hsa-mir-371, hsa-mir-526b, hsa-mir-516b-1, hsa-mir-516b-2, hsa-mir-521-2, hsa-mir-521-1, hsa-mir-516a-1, hsa-mir-498, hsa-mir-516a-2, hsa-mir-935, hsa-mir-517b, hsa-mir-517a, hsa-mir-517c | |
| chr20:14,986,306-14,993,757 | Loss | 7,452 | p12.1 | MACROD2 | | |
| chr22:35,546,170-35,687,378 | Loss | 141,209 | q12.3 | CSF2RB, NCF4 | | |

| Chromosome Region (hg18) | Event | Length (Kb) | Cytoband | Genes | miRNAs | Cancer Genes (Cancer Gene Census) |
|---|---|---|---|---|---|---|
| chrX:918,399-921,332 | Loss | 2,934 | p22.33 | | | |
| chrX:2,803,844-3,734,485 | Loss | 930,642 | p22.33 | ARSD, ARSE, ARSF, ARSH, CXorf28, GYG2, MXRA5, PRKX | | |
| chrX:55,950,543-55,967,087 | Loss | 16,545 | p11.21 | | | |
| chrX:90,356,371-92,252,215 | Loss | 1895,845 | q21.31 - q21.32 | PABPC5, PCDH11X | | |
| chrY:918,399-926,844 | Loss | 8,446 | p11.32 | | | |
| chrY:7,489,021-7,711,787 | Gain | 222,767 | p11.2 | TTTY16 | | |
| chrY:17,030,541-26,689,998 | Gain | 9659,458 | q11.221 - q11.23 | BCORP1, BPY2, BPY2B, BPY2C, CD24, CDY1, CDY1B, CDY2A, CDY2B, CSPG4P1Y, DAZ1, DAZ2, DAZ3, DAZ4, EIF1AY, FAM224A, FAM224B, FAM41AY1, FAM41AY2, GOLGA2P2Y, GOLGA2P3Y, HSFY1, HSFY2, KDM5D, NCRNA00185, PRY, PRY2, RBMY1A1, RBMY1B, RBMY1D, RBMY1E, RBMY1F, RBMY1J, RBMY2EP, RBMY2FP, RPS4Y2, TTTY10, TTTY13, TTTY14, TTTY17A, TTTY17B, TTTY17C, TTTY3, TTTY3B, TTTY4, TTTY4B, TTTY4C, TTTY5, TTTY6, TTTY6B, TTTY9A, TTTY9B, TXLNG2P, XKRY, XKRY2 | | |

**Table S6.** Variations recurrently identified in array-CGH experiments (Sup Table S3, F3 *vs* E5; Sup Table S4, A23 *vs* C9; Sup Table S5, A24 *vs* B4).

| Chromosome Region | Length (Kb) | Cytoband | Gene symbol or miRNA | Name | Biological Process |
|---|---|---|---|---|---|
| chr1:116,337,471-116,910,628 | 573,158 | p13.1 | SLC22A15 | solute carrier family 22, member 15 | ion transport |
| | | | MAB21L3 | mab-21-like 3 (C. elegans) | - |
| | | | ATP1A1 | ATPase, Na+/K+ transporting, alpha 1 polypeptide | ATP biosynthetic process, ion transmembrane transport, negative regulation of glucocorticoid biosynthetic process, regulation of heart contraction, positive regulation of striated muscle contraction, regulation of blood pressure, regulation of sodium ion transport, regulation of the force of heart contraction, response to drug, transmembrane transport |
| | | | ATP1A1OS | ATP1A1 opposite strand | - |
| | | | CD58 | CD58 molecule | blood coagulation, cell adhesion, cell-cell adhesion, leukocyte migration |
| chr1:229,220,576-229,446,737 | 226,162 | q42.2 | MIR1182 | microRNA 1182 | MicroRNA expression signature of human sarcomas |
| | | | FAM89A | family with sequence similarity 89, member A | - |
| | | | LOC149373 | uncharacterized LOC149373 | - |
| | | | TRIM67 | tripartite motif containing 67 | zinc ion binding |
| | | | C1orf131 | chromosome 1 open reading frame 131 | - |
| | | | GNPAT | glyceronephosphate O-acyltransferase | cellular lipid metabolic process, cellular membrane organization, cerebellum morphogenesis, ether lipid biosynthetic process, glycerophospholipid biosynthetic process, paranodal junction assembly, phosphatidic acid biosynthetic process, phospholipid metabolic process, response to drug, response to fatty acid, response to nutrient, response to starvation, small molecule metabolic process, synapse assembly |

| Chromosome Region | Length (Kb) | Cytoband | Gene symbol or miRNA | Name | BiologicalProcess |
|---|---|---|---|---|---|
| chr3:173,711,933-174,194,891 | 482,959 | q26.31 | TNFSF10 | tumor necrosis factor (ligand) superfamily, member 10 | activation of cysteine-type endopeptidase activity involved in apoptotic process, cell-cell signaling, immune response, positive regulation of I-kappaB kinase/NF-kappaB cascade, positive regulation of extrinsic apoptotic signaling pathway, positive regulation of release of cytochrome c from mitochondria, signal transduction |
| | | | NCEH1 | neutralcholesterol ester hydrolase 1 | lipidcatabolicprocess |
| | | | ECT2 | epithelial cell transforming sequence 2 oncogene | activation of RacGTPase activity, activation of Rho GTPase activity, activation of protein kinase activity, apoptotic process, cell cycle cytokinesis, cell morphogenesis, cellular response to calcium ion, cellular response to hydrogen peroxide, cellular response to ionizing radiation, nerve growth factor receptor signaling pathway, positive regulation of Cdc42 GTPase activity, positive regulation of I-kappaB kinase/NF-kappaB cascade, positive regulation of Rho GTPase activity, positive regulation of Rho GTPase activity, positive regulation of neuron differentiation, positive regulation of protein import into nucleus, protein homooligomerization, protein transport, regulation of attachment of spindle microtubules to kinetochore, tight junction assembly |
| | | | SPATA16 | spermatogenesisassociated 16 | cell differentiation, multicellular organismal development, spermatogenesis |
| chr8:130,637,003-130,770,082 | 133,08 | q24.21 | - | - | - |
| chr9:2,496,958-2,523,497 | 26,54 | p24.2 | - | - | - |
| chr9:32,004,034-32,102,682 | 98,649 | p21.1 | - | - | - |
| chr9:5,380,368-5,999,912 | 619,545 | p24.1 | PLGRKT | plasminogenreceptor, C-terminal lysine transmembraneprotein | chemotaxis, inflammatory response, positive regulation of plasminogen activation |
| | | | CD274 | CD274 molecule | T cellcostimulation, cellproliferation, cell surface receptorsignalingpathway, immune response, negativeregulation of T cellproliferation, signal transduction |
| | | | PDCD1LG2 | programmedcelldeath 1 ligand 2 | T cellcostimulation, immune response, regulation of T cellproliferation |
| | | | KIAA1432 | KIAA1432 | |
| | | | ERMP1 | endoplasmicreticulum metallopeptidase 1 | ovarianfollicledevelopment, proteolysis |
| | | | MLANA | melan-A | proteinbinding |
| | | | KIAA2026 | KIAA2026 | - |
| | | | MIR4665 | microRNA 4665 | - |

| Chromosome Region | Length (Kb) | Cytoband | Gene symbol or miRNA | Name | BiologicalProcess |
|---|---|---|---|---|---|
| **chrY:22,636,421-23,058,870** | 422,45 | q11.223 | PRY | PTPN13-like, Y-linked | - |
| | | | PRY2 | PTPN13-like, Y-linked 2 | - |
| | | | TTTY6 | testis-specifictranscript, Y-linked 6 (non-proteincoding) | - |
| | | | TTTY6B | testis-specifictranscript, Y-linked 6B (non-proteincoding) | - |
| | | | RBMY1F | RNA binding motif protein, Y-linked, family 1, member F | - |
| | | | RBMY1J | RNA binding motif protein, Y-linked, family 1, member J | RNA splicing, mRNA processing, spermatogenesis |
| | | | TTTY5 | testis-specifictranscript, Y-linked 5 | - |
| | | | RBMY2FP | RNA binding motif protein, Y-linked, family 2, member F pseudogene | - |
| **chrY:23,171,649-23,327,051** | 155,403 | q11.223 | - | - | - |
| **chrY:26,659,439-26,689,998** | 30,56 | q11.23 | - | - | - |
